# SARS-CoV-2 Delta and Omicron variants evade population antibody response by mutations in a single spike epitope

**DOI:** 10.1038/s41564-022-01235-4

**Published:** 2022-09-23

**Authors:** Ping He, Banghui Liu, Xijie Gao, Qihong Yan, Rongjuan Pei, Jing Sun, Qiuluan Chen, Ruitian Hou, Zimu Li, Yanjun Zhang, Jincun Zhao, Hao Sun, Bo Feng, Qian Wang, Haisu Yi, Peiyu Hu, Pingchao Li, Yudi Zhang, Zhilong Chen, Xuefeng Niu, Xiaolin Zhong, Liang Jin, Xiaofeng Liu, Kun Qu, Katarzyna A. Ciazynska, Andrew P. Carter, John A. G. Briggs, Jizheng Chen, Jinsong Liu, Xinwen Chen, Jun He, Ling Chen, Xiaoli Xiong

**Affiliations:** 1 State Key Laboratory of Respiratory Disease, CAS Key Laboratory of Regenerative Biology, Guangdong Provincial Key Laboratory of Stem Cell and Regenerative Medicine, Guangdong Provincial Key Laboratory of Biocomputing, Guangzhou Institutes of Biomedicine and Health, Chinese Academy of Sciences, Guangzhou, China; 2 University of Chinese Academy of Science, Beijing, China; 3 Bioland Laboratory (Guangzhou Regenerative Medicine and Health - Guangdong Laboratory), Guangzhou, China; 4 State Key Laboratory of Respiratory Disease, Guangzhou Institute of Respiratory Health, First Affiliated Hospital of Guangzhou Medical University, Guangzhou, China; 5 State Key Laboratory of Virology, Wuhan Institute of Virology, Center for Biosafety Mega-Science, Chinese Academy of Sciences, Wuhan, China; 6 Guangzhou Eighth People’s Hospital, Guangzhou Medical University, Guangzhou, China; 7 School of Biomedical Sciences, Huaqiao University, Quanzhou, China; 8 Xiamen United Institute of Respiratory Health, Xiamen, China; 9 Angitia Biopharmaceuticals, Guangzhou, China; 10 Infectious Diseases Translational Research Programme, Department of Biochemistry, Yong Loo Lin School of Medicine, National University of Singapore, Singapore, Singapore; 11 Structural Studies Division, Medical Research Council Laboratory of Molecular Biology, Cambridge, UK; 12 Max Planck Institute of Biochemistry, Martinsried, Germany; 13 Guangzhou Laboratory, Guangzhou International Bio Island, Guangzhou, Guangdong Province China

**Keywords:** Viral infection, SARS-CoV-2, Cryoelectron microscopy, Immune evasion

## Abstract

Population antibody response is thought to be important in selection of virus variants. We report that SARS-CoV-2 infection elicits a population immune response that is mediated by a lineage of VH1-69 germline antibodies. A representative antibody R1-32 from this lineage was isolated. By cryo-EM, we show that it targets a semi-cryptic epitope in the spike receptor-binding domain. Binding to this non-ACE2 competing epitope results in spike destruction, thereby inhibiting virus entry. On the basis of epitope location, neutralization mechanism and analysis of antibody binding to spike variants, we propose that recurrent substitutions at 452 and 490 are associated with immune evasion of the identified population antibody response. These substitutions, including L452R (present in the Delta variant), disrupt interactions mediated by the VH1-69-specific hydrophobic HCDR2 to impair antibody-antigen association, enabling variants to escape. The first Omicron variants were sensitive to antibody R1-32 but subvariants that harbour L452R quickly emerged and spread. Our results provide insights into how SARS-CoV-2 variants emerge and evade host immune responses.

## Abstract (web summary)

Amino acid changes in a newly identified spike epitope enabled the Delta variant to evade a population antibody response.

## Main

Owing to immune pressure induced by natural infection and vaccination, numerous SARS-CoV-2 variants have emerged, these variants encoding spike proteins with substituted amino acids that function to evade antibody neutralization^1^. Several recurrent receptor-binding domain (RBD) substitutions have been observed among variants: E484K was found in the Beta (B.1.351), Eta (B.1.525), Iota (B.1.526) and Gamma (P.1/ P.1.1/ P.1.2) variants; N501Y was first found in the Alpha (B.1.1.7) variant and subsequently found in the Beta and Gamma variants associated with recurrent K417N/T changes; L452R was found in the Epsilon (B.1.427/B.1.429), Kappa (B.1.617.1), Delta (B.1.617.2) and B.1.617.3 variants. On the basis of epitope locations, RBD-targeting antibodies have been grouped into 4 classes^2^. Recurrent substitutions at 484 and 417/501 enable evasion of VH1-2 class 2 and VH3-53/3-66 class 1 RBD antibodies, respectively. These antibodies have germline-like sequences and are widely present in the population, representing two distinct shared antibody responses^3,4^. Interestingly, although a shared antibody response escaped by the recurrent substitution L452R has remained unidentified, the L452R-bearing Delta variant, despite lacking substitutions at 484 and 417/501, displaced the Alpha, Beta, Gamma, Kappa and B.1.617.3 variants to become globally dominant^5^. It is currently not fully understood what evolutionary advantage the Delta variant had compared with other variants. The original Omicron BA.1 (B.1.1.529) variant is highly mutated, containing E484A, K417N/N501Y and numerous other substitutions, but interestingly, it has no substitution at 452.

Here we report identification of a population immune response to SARS-CoV-2 and discuss how this relates to the emergence of L452R-bearing variants of concern including Delta and Omicron BA.4/BA.5 variants.

## Results

### Antibody R1-32 neutralizes SARS-CoV-2 variants including Omicron

**Fig. 1:**
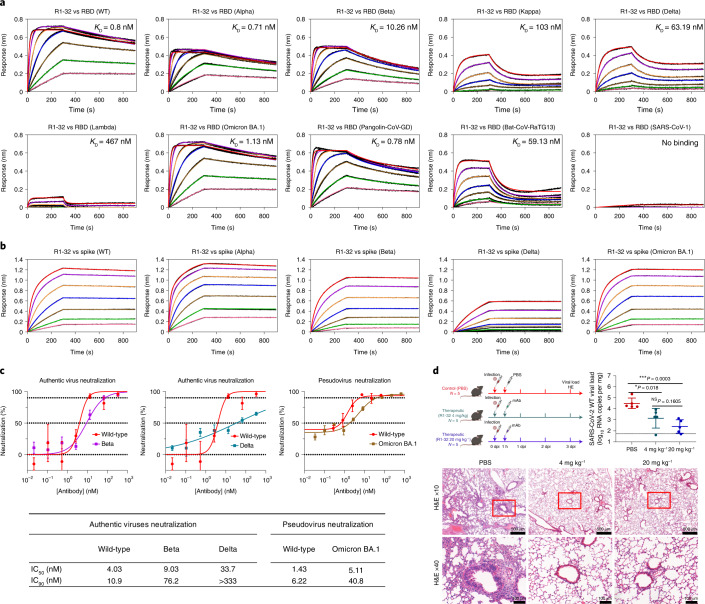
In vitro and in vivo activities of R1-32. **a**, Binding of R1-32 to wild-type, variant SARS-CoV-2, animal-origin SARS-related CoV and SARS-CoV-1 RBDs. **b**, Binding of R1-32 to wild-type and selected variant SARS-CoV-2 spikes. BLI binding assays used 2-fold RBD or spike dilution series (200 nM to 3.125 nM); fitted kinetic parameters are summarized in Supplementary Table 1. **c**, Top: plaque reduction neutralization activities of R1-32 (mean ± s.e.m.) towards selected variant viruses compared to neutralization towards the wild-type virus. *n* = 3, except for neutralization of wild-type pseudovirus, *n* = 2. Representative data are shown from at least 2 independent experiments. Bottom: summary of IC_90_ and IC_50_ values. **d**, Top left: animal protection experiment design, created with BioRender.com. R1-32 (4 mg kg^−1^ or 20 mg kg^−1^ of body weight) was intraperitoneally (i.p.) injected into hACE2 transgenic mice 1 h after SARS-CoV-2 infection. PBS injections were used for the control group. Top right: virus titres in lung tissues were determined at 3 dpi by RT-qPCR (*n* = 5). Data are presented as mean ± s.d. The *P* values were determined by two-sided unpaired *t*-test. *P < 0.05, ***P < 0.001; NS, not significant. Bottom: histopathology analysis of lung tissues at 3 dpi. The images and areas of interest are ×10 (scale bar, 500 µm) and ×40 (scale bar, 100 µm). Dotted lines represent limits of detection. Source data

**Extended Data Fig. 1:**
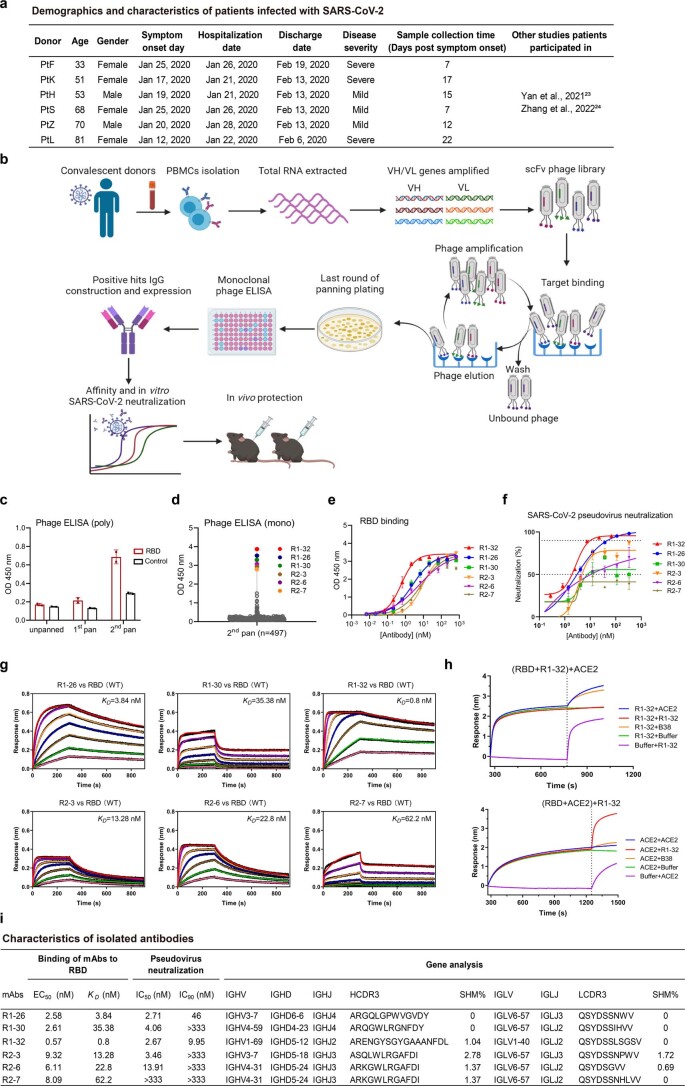
Isolation and characterization of antibodies. **a**, Demographics of study subjects. PBMCs were collected from 6 confirmed COVID-19 patients in hospital at a mean duration of 14 (±7) days after symptom onset. **b**, Construction and screening of phage antibody display libraries. A total of 6 convalescent patients’ PBMCs were collected. The 6 scFv phage libraries displaying the variable regions of antibody heavy chain (VH) and light chain (VL) were established from the individual convalescent patient’s PBMC. The phage libraries screening used the SARS-CoV-2 RBD recombinant protein as a bait. Based on monoclonal phage ELISA, positive binding clones of single phage were sequenced and the pairs of VH/VL were reconstructed into human IgG1 antibodies for expression. Finally, the purified antibodies were functionally verified. **c**, Polyclonal phage ELISA binding activities (mean ± SD) of the unpanned, 1^st^ panned and 2^nd^ panned phage libraries using SARS-CoV-2 RBD. Control used an unrelated antigen. Data are shown from a single experiment with 2 replicates. **d**, The 6 strongest binding single phages were colored among the 497 reactive clones from the 2^nd^ panning. **e**, RBD binding activities (mean ± SD) of the 6 isolated monoclonal antibodies (mAbs) by ELISA. **f**, Neutralization activities (mean ± SEM) of the 6 mAbs against SARS-CoV-2 pseudovirus. Data are shown from a single experiment with 3 replicates (**e**, **f**). **g**, Binding kinetics of six candidate antibodies to the SARS-CoV-2 RBD were measured by biolayer interferometry (BLI) with antibody immobilized on the biosensor and RBD in solution. The black lines represent the experimentally recorded sensorgram traces, the colored lines represent fittings to the sensorgram traces. A 2-fold RBD dilution series (200 nM to 3.125 nM) were used in the BLI assays, detailed binding kinetics parameters were summarized in Supplementary Table 5. **h**, Competitive binding between R1-32 and ACE2 to the SARS-CoV-2 RBD as assessed by BLI. Biosensors immobilized with SARS-CoV-2 RBD were first saturated with R1-32 (upper panel) or ACE2 (lower panel) as indicated by the dashed lines, and then dipped into R1-32 (red) or ACE2 (blue) solutions of the same concentration. An ACE2 competing antibody B38 (orange) or buffer (green) were used as controls. Controls without binding of the first ligand were also included (purple). **i**, Characteristics of isolated antibodies. EC_50_ and *K*_*D*_ of antibody binding to SARS-CoV-2 RBD measured by ELISA (data shown in **e**) and BLI (data shown in **g**) respectively were summarized. Sequence analysis of antibodies was performed by IMGT. Source data

**Extended Data Fig. 2:**
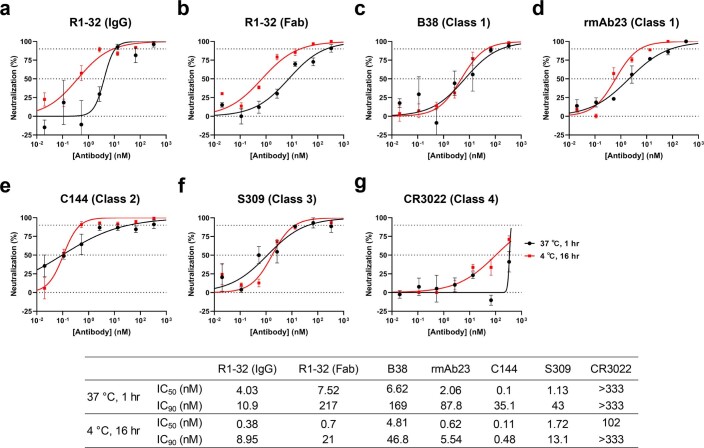
Neutralization of authentic wild-type virus by R1-32 compared to selected antibodies of different RBD targeting antibody classes. **a-g**, Neutralization activities of wild-type virus (Wuhan strain) by **a**, R1-32 IgG, **b**, R1-32 Fab, **c**, B38 (ref. ^59^), **d**, rmAb23 (ref. ^23^), **e**, C144 (ref. ^2^), **f**, S309 (ref. ^60^), **g**, CR3022 (ref. ^61^) were evaluated by plaque reduction neutralization assays. Selected antibodies of different RBD antibody classes were included for comparison (see Extended Data Fig. 7 and Supplementary Fig. 3 for their classification and epitopes). Viruses were incubated with antibodies for standard 1 hr duration (black data points) or an extended duration of 16 hr (red data points) before inoculation (data points and error bars represent mean ± SEM). *n* = 3. Representative data from at least 2 independent experiments are shown. IC_90_ and IC_50_ values are summarized in the table below. Prolonged incubation improved R1-32 IgG neutralization likely due to more saturated R1-32 binding to spike. Source data

First, we isolated 6 antibodies with high affinity for spike RBD binding, by phage display of antibody genes derived from peripheral blood mononuclear cells (PBMCs) of 6 COVID-19 convalescent patients infected with SARS-CoV-2 in January 2020 (Extended Data Fig. 1a–e). The strongest RBD binder, a VH1-69 antibody that we named R1-32, exhibited the strongest pseudovirus neutralizing activity (IC_90_ = 9.95 nM), without inhibiting spike ACE2 binding (Extended Data Fig. 1f–i). Biolayer interferometry (BLI) assays showed that R1-32 binds to wild-type SARS-CoV-2 RBD with high affinity (*K*_D_ = 0.8 nM) and maintains high-affinity binding to the RBDs of the Alpha (*K*_D_ = 0.71 nM), Beta (*K*_D_ = 10 nM) and Omicron BA.1 (*K*_D_ = 1 nM) variants (Fig. 1a**)**. Binding to the RBDs of the Kappa (*K*_D_ = 103 nM), Delta (*K*_D_ = 63 nM) and Lambda (*K*_D_ = 467 nM) variants was greatly reduced (Fig. 1a). In addition, R1-32 showed some cross reactivity by binding to the RBDs of the Guangdong (GD) pangolin (*K*_D_ = 0.78 nM) and the RaTG13 bat (*K*_D_ = 59 nM) SARS-related CoV spikes (Fig. 1a). Consistent with these RBD binding data, R1-32 binding to the Delta variant spike trimer was greatly reduced, whereas binding to spike trimers of the other tested variants remained tight and non-dissociating, similar to binding to the wild-type virus (Fig. 1b). Also consistent with the binding data: R1-32 exhibited comparable or better neutralization activity towards wild-type SARS-CoV-2 authentic virus compared with other RBD antibodies already characterized (Fig. 1c and Extended Data Fig. 2); and it maintained good neutralization of the Beta variant authentic virus and the Omicron BA.1 pseudovirus (Fig. 1c). Neutralization of the Delta variant authentic virus was greatly abrogated (Fig. 1c). In a human ACE2 transgenic mouse model^6^, intraperitoneal administrations of R1-32 at 4 mg kg^−1^ and 20 mg kg^−1^ at 1 h post intranasal inoculation of 5 × 10^5^ plaque-forming units (p.f.u.) SARS-CoV-2 wild-type virus were able to significantly reduce viral load in lung, 3 d post infection, compared with the control group (Fig. 1d). There was clear indication of reduced lung inflammation in the mice protected by R1-32 (Fig. 1d). These results establish that R1-32 has protection activity towards SARS-CoV-2 infection.

### R1-32 binding promotes RBD opening

**Fig. 2:**
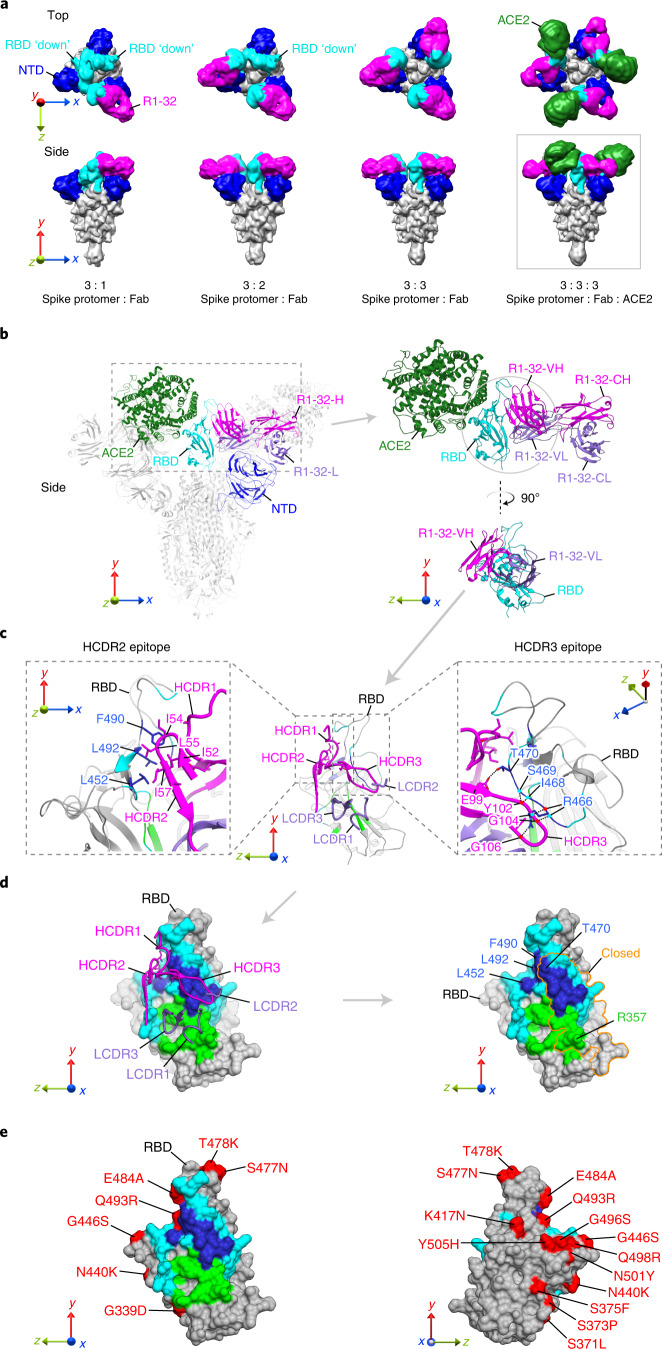
Structures of R1-32 in complex with SARS-CoV-2 spike and R1-32 epitope. **a**, Cryo-EM densities (low-pass filtered to 10 Å) of S-GSAS/6P spikes bound to R1-32 Fab and ACE2 at different stoichiometries. R1-32 Fab, NTD, RBD and ACE2 are highlighted in magenta, blue, cyan and dark green, respectively; the rest of the spike is coloured grey. RBDs adopting ‘down’ positions are indicated. **b**, Structure of the 3:3:3 spike protomer:Fab:ACE2 complex derived from the boxed density in **a**. R1-32-H and R1-32-L are coloured in magenta and purple. Structure of the RBD:Fab:ACE2 portion is shown on the right panels. **c**, R1-32 HCDR2 and HCDR3 epitopes are shown with rotations in viewing angles. Residues interacting with R1-32-H and R1-32-L are coloured in cyan and green, respectively. Highly buried residues (buried surface area >70% of accessible surface area) are coloured blue to highlight the HCDR2 and HCDR3 epitopes. R1-32 HCDR2 interacts with epitopes by hydrophobic contacts (left panel). R1-32 HCDR3 primarily uses backbone carbonyl oxygens and amide nitrogens (indicated by red and cyan dots, respectively) to hydrogen bond with epitope (right panel). **d**, Left: surface representation of the R1-32 epitope, with surface areas coloured as in **c**. Right: epitope area within the orange outline is buried by NTD in an ‘RBD’ down spike protomer. **e**, Epitopes of R1-32 are coloured as in **d** and substituted residues in the Omicron BA.1 variant are highlighted in red.

**Extended Data Fig. 3:**
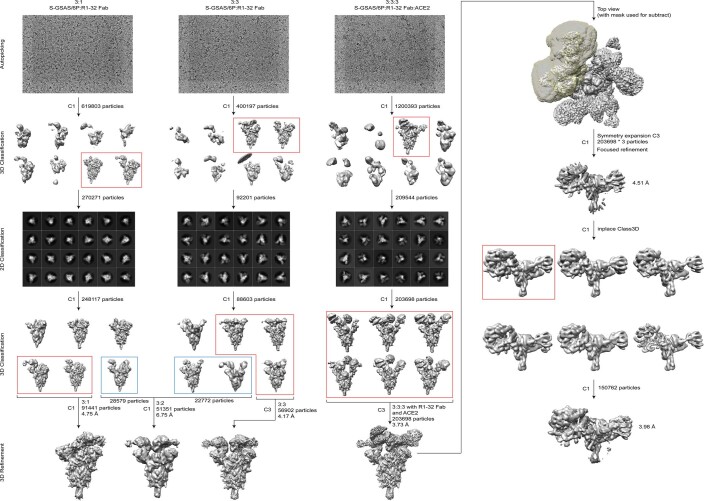
Cryo-EM data processing flow-chart. Pipelines are illustrated for the 3:1, 3:3 spike protomer:R1-32 Fab and the 3:3:3 spike protomer:R1-32 Fab:ACE2 datasets. After automated picking, 3D and 2D classification steps were used to remove contaminating objects. 3D classification was subsequently used to sort the data into different structures. The structure of the RBD:R1-32 Fab:ACE2 portion was further improved with focused refinement centred at the RBD.

To further understand the R1-32 neutralization mechanism, we determined cryo-electron microscopy (cryo-EM) structures of spike:R1-32 Fab complexes in three different stoichiometries, as well as structures of spike:R1-32 Fab:ACE2 complex, with resolutions of 3.73–6.75 Å (Extended Data Figs. 3 (from left to right) and 4c–g, and Supplementary Table 2). These structures show that R1-32 Fab approaches from outside of the spike above the N-terminal domain (NTD) to bind the RBD. The bound Fab adopts an orientation perpendicular to the spike 3-fold axis (Fig. 2a). The protomers bound by R1-32 adopt an open conformation with RBDs in an ‘up’ position. The unbound protomers adopt a closed conformation with RBDs in a ‘down’ position (Fig. 2a). By varying the amount of Fab added (3:1 and 3:3 spike protomer:R1-32 Fab molar ratios), we observed 3:1, 3:2 and 3:3 spike protomer:R1-32 Fab complexes (Fig. 2a and Extended Data Fig. 3), suggesting that R1-32 binding opens spike RBD stoichiometrically. The 3:3:3 spike protomer:R1-32 Fab:ACE2 complex structure confirmed that R1-32 does not block ACE2 binding (Fig. 2a).

### R1-32 uses HCDR2 and HCDR3 to bind its epitope

**Extended Data Fig. 4:**
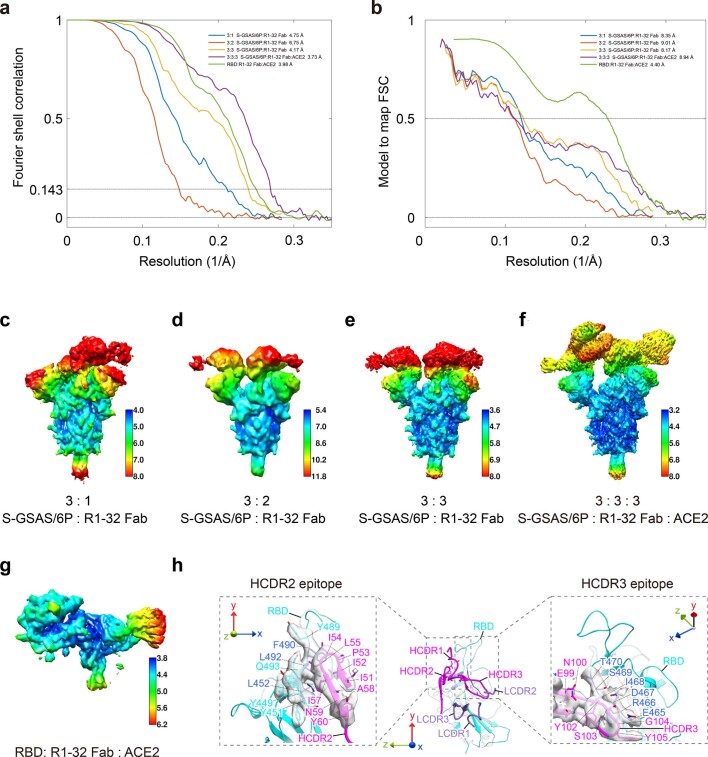
Resolution assessment of cryo-EM structures. **a**, Global resolution assessment by Fourier shell correlation at the 0.143 criterion. **b**, Correlations of model vs map by Fourier shell correlation (FSC) at the 0.5 criterion. **c-g**, Local resolution maps for all structures calculated using RELION. **h**, Representative densities of the R1-32 epitopes and R1-32 HCDR loops in the focused refinement map. R1-32 HCDRs, LCDRs and RBD are colored in magenta, purple and cyan, respectively.

**Extended Data Fig. 5:**
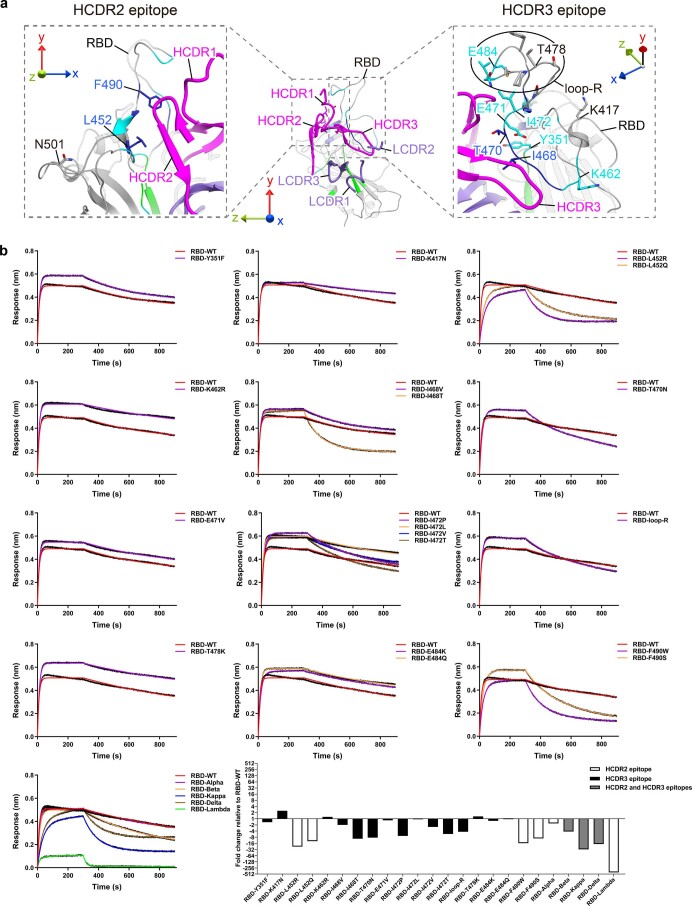
Effects of mutations on R1-32 binding. **a**, Locations of substituted residues are shown in the context of the R1-32 epitope, loop-R indicates replacement of SARS-CoV-2 ACE2 binding loop (474-486) to the corresponding SARS-CoV-1 residues (461-472). **b**, Binding of R1-32 to 24 substituted RBDs were assayed by BLI and compared to the wild-type (WT) RBD. Assays were carried out at a fixed RBD concentration of 200 nM. Kinetic parameters are summarized in Supplementary Table 6, fold changes of *K*_*D*_ values were normalized to WT on a 2^n^ scale (the last panel).

On the basis of a 3.8 Å resolution RBD-centred focused refined map derived from the 3:3:3 spike protomer:R1-32 Fab:ACE2 complex dataset (Extended Data Fig. 4g,h), we built an atomic model revealing side-chain interactions between RBD and R1-32 Fab. Our model revealed that R1-32 and ACE2 bind to two opposite, non-overlapping molecular surfaces on an RBD (Fig. 2b), consistent with R1-32 being non-inhibitory to ACE2 binding. R1-32 binding buried an epitope area of 1,214 Å^2^ on RBD, 813 Å^2^ and 401 Å^2^ of which are buried by HCDRs and LCDRs, respectively (Fig. 2c,d). HCDR2 and HCDR3 of R1-32 primarily mediated interactions with RBD (Fig. 2c,d). The HCDR2 epitope contains hydrophobic residues L452, F490 and L492; this epitope was recognized by the hydrophobic R1-32 HCDR2 residues I52, I54, L55 and I57 via hydrophobic interaction (Fig. 2c, left); R1-32 binds with greatly reduced affinities to RBDs with substitutions in the HCDR2 epitope (Kappa (L452R/E484Q), Delta (L452R/T478K), Lambda (L452Q/F490S), RaTG13 (F490Y), SARS-CoV-1 (L452K/F490W)) (Fig. 1a and Supplementary Fig. 1), suggesting that 452 and 490 within the HCDR2 epitope are extremely important for R1-32 binding. We further confirmed this suggestion with point mutation data (Extended Data Fig. 5). HCDR2 being hydrophobic is the defining feature of VH1-69 antibodies and HCDR2 residues are known to be polymorphic. In the case of R1-32, L55 (L54 in Kabat numbering) facilitates better RBD binding than a phenylalanine (F), which is also common in the population and has been shown to play important roles in generating broadly neutralizing antibodies against diverse viruses^7^ (Supplementary Fig. 2). The HCDR3 epitope contains mostly polar residues—R466, I468, S469 and T470; this epitope interacts with HCDR3 via extensive hydrogen bonding (Fig. 2c, right). Point mutation data suggest that substitutions within the HCDR3 epitope have weaker effect on R1-32 binding (Extended Data Fig. 5).

### R1-32 targets a spike-destructing semi-cryptic epitope

**Fig. 3:**
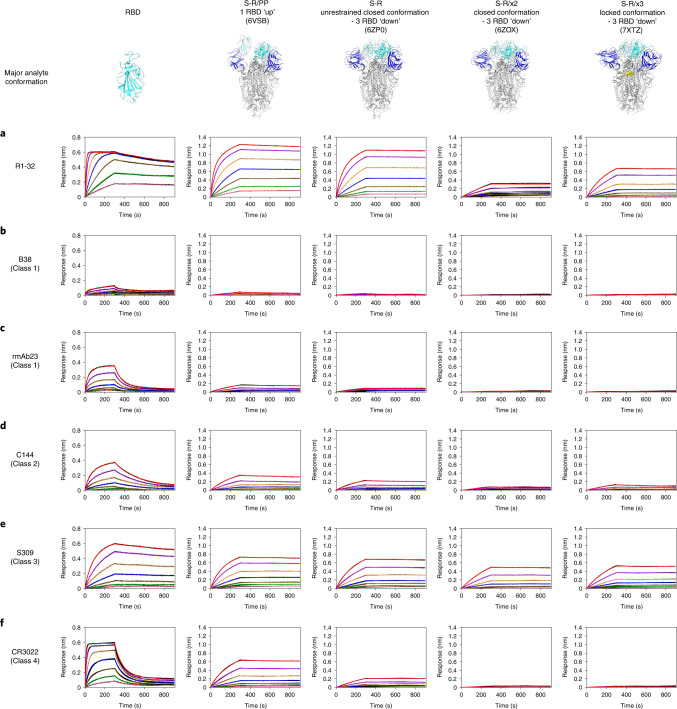
Binding of R1-32 and selected antibodies of different RBD antibody classes to SARS-CoV-2 RBD and spikes in different conformations. **a**–**f**, Binding of R1-32 (**a**), B38 (ref. ^59^), (**b**), rmAb23 (ref. ^23^), (**c**), C144 (ref. ^2^), (**d**), S309 (ref. ^60^) (**e**) and CR3022 (ref. ^61^) (**f**) from different RBD-targeting antibody classes (see Extended Data Fig. 7 and Supplementary Fig. 3 for their classification and epitopes) to SARS-CoV-2 RBD and spike trimers of different conformations. Characterized previously, major conformations of S-R/PP, S-R, S-R/x2 and S-R/x3 spikes used as analytes in the BLI assays are shown in the top row. S-R/PP (2P-stabilized spike with furin site changed to a single R) and S-R (unstabilized native spike with furin site changed to a single R) have been shown to exist primarily in 1 RBD ‘up’ (80% 1 RBD ‘up’, 20% closed) and unrestrained closed (20% 1 RBD ‘up’, 80% closed) conformations, respectively^8^. RBDs in S-R/x2 and S-R/x3 spikes were restrained by the x2 and x3 disulfide bonds in ‘down’ positions, and the two spikes differ slightly in trimer packing, adopting primarily closed^8^ and locked^42^ conformations, respectively. IgGs were immobilized onto Protein A biosensors and submerged into 2-fold serially diluted RBD and spike solutions (200, 100, 50, 25, 12.5, 6.25, 3.125 nM) to record sensorgrams (black lines). The fits of the data are shown as coloured lines. Rate constants (*k*_on_ and *k*_off_) estimated from the association and dissociation phases and dissociation constants (*K*_D_) derived from kinetic analyses are summarized in Supplementary Table 3.

**Fig. 4:**
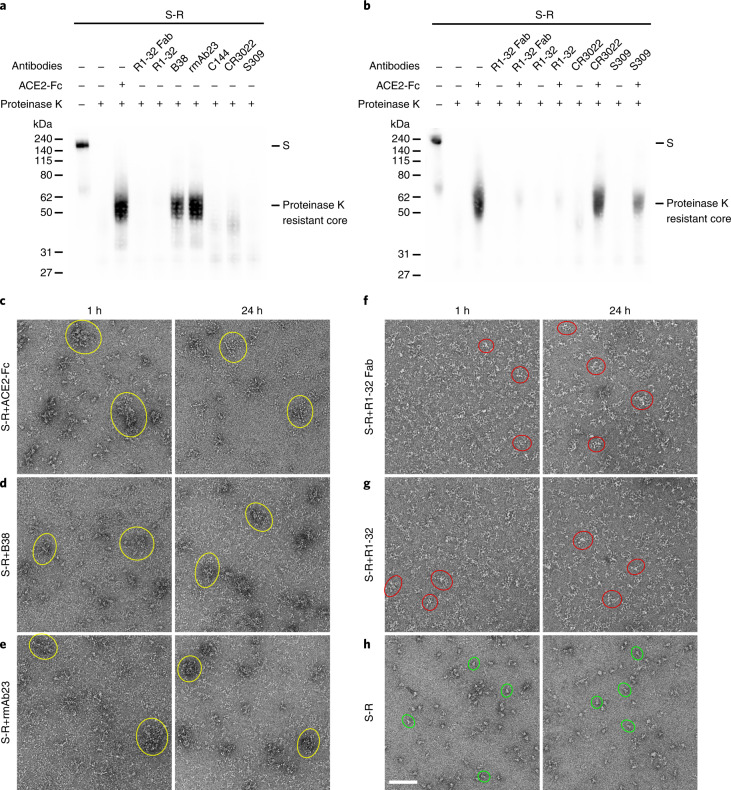
R1-32 binding resulted in destruction of SARS-CoV-2 spike structure. **a**,**b**, Ligand-induced conformational change assays to probe the induction of post-fusion structures. **a**, Native spike (S-R) (7.09 µM) was incubated with molar excess of ACE2-Fc or antibodies for 1 h. Samples were analysed by western blotting to detect generation of the 55 kDa proteinase K-resistant core, which is a signature of the post-fusion S2 structure^62^. ACE2-Fc and class 1 antibodies B38 and rmAb23 induced post-fusion structures. **b**, Inhibition of conformational change by non-ACE2 competing antibodies were assayed by antibody pre-incubation (1 h) before further incubation (1 h) with ACE2-Fc. Only R1-32 was able to abolish fusogenic spike conformational change among the tested non-ACE2 competing antibodies. Representative data are shown from at least 3 independent experiments (**a**,**b**). The effects of ACE2-Fc and antibody binding on the structure of SARS-CoV-2 S-R spike after 1 h or 24 h incubations were assessed by negative-stain EM. **c**–**e**, Binding of ACE2-Fc (**c**), B38 (**d**) and rmAb23 (**e**) induced post-fusion structures that aggregate to form rosettes (examples are outlined by yellow circles) similar to our previous observations on the SARS-CoV-1 spike^63^. **f**,**g**, Binding of R1-32 Fab (**f**) or R1-32 (**g**) IgG to the S-R spike caused the spike to disintegrate into smaller structures (examples are outlined by red circles). **h**, Images for the S-R spike in the control experiment in which no ligand was added (intact spike examples are outlined by green circles). Scale bar, 100 nm. Representative data are shown from at least 2 independent experiments (**c**–**h**). Source data

**Extended Data Fig. 6:**
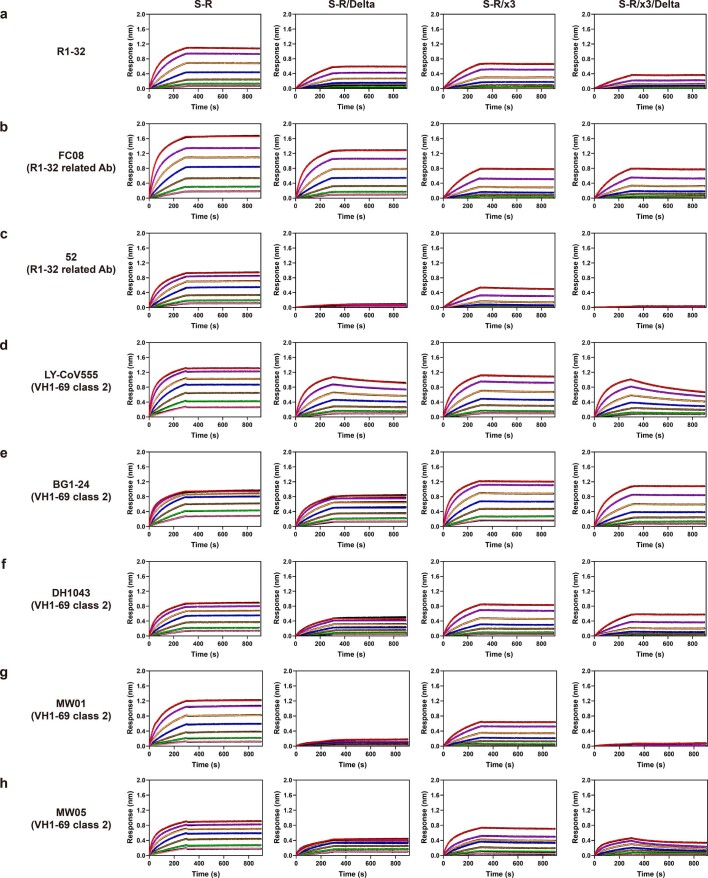
Comparison of VH1-69 antibody binding to Delta variant and wild-type spikes with or without x3 disulfide. **a**, VH1-69 antibody R1-32; R1-32 related VH1-69 antibodies **b**, FC08, **c**, 52; selected class 2 VH1-69 antibodies **d**, LY-CoV555, **e**, BG1-24, **f**, DH1043, **g**, MW01, **h**, MW05 were immobilized onto Protein A biosensors. Bindings of antibodies to unstabilized native (S-R) (ref. ^8^) and x3 disulfide stabilized RBD ‘down’ (S-R/x3) spikes (ref. ^42^) were compared to the corresponding Delta variant spikes (S-R/Delta, S-R/x3/Delta). Kinetic parameters are summarized in Supplementary Table 7.

The R1-32 epitope is partially buried when RBD adopts a ‘down’ position: only the HCDR2 epitope is fully exposed in RBD ‘down’ spikes; areas bound by HCDR3, LCDR1, LCDR2 and LCDR3 are buried to different extents by the NTD of a neighbouring protomer when RBD is ‘down’ (Fig. 2d). In particular, the HCDR3 epitope is almost completely buried when RBD is ‘down’ (Fig. 2d, right). This semi-cryptic epitope explains why R1-32 binding promotes RBD to adopt an ‘up’ position. We found that R1-32 was able to bind disulfide-stabilized RBD ‘down’ spikes^8^ with high affinity without dissociating (Fig. 3). This process probably disrupted the stabilized spike trimers, suggesting that the unhindered HCDR2 epitope can serve as a hydrophobic anchor strong enough to disrupt the stabilized RBD ‘down’ spikes. We further found that unstabilized native spike pre-incubated with R1-32 became unable to undergo structural transition into a post-fusion conformation (Fig. 4a**)**. By negative-stain EM, we found that native spike incubated with R1-32 disintegrated into smaller structures (Fig. 4f,g), suggesting that R1-32 binding generated labile open spike structures that disintegrate. Taken together, we conclude that R1-32 most probably neutralizes virus by destruction of the spike structure to inhibit virus cell entry. Consistent with this mechanism, substitutions at 452 and 490, which strongly hinder R1-32 association with RBD (with slower kinetics and lower responses in the association stages) (Fig. 1a,b and Extended Data Fig. 6a), are likely to result in unsaturated spike binding, rendering R1-32 neutralization ineffective, as observed for the Delta variant (Fig. 1c).

### R1-32 represents a distinct RBD-targeting antibody class

**Fig. 5:**
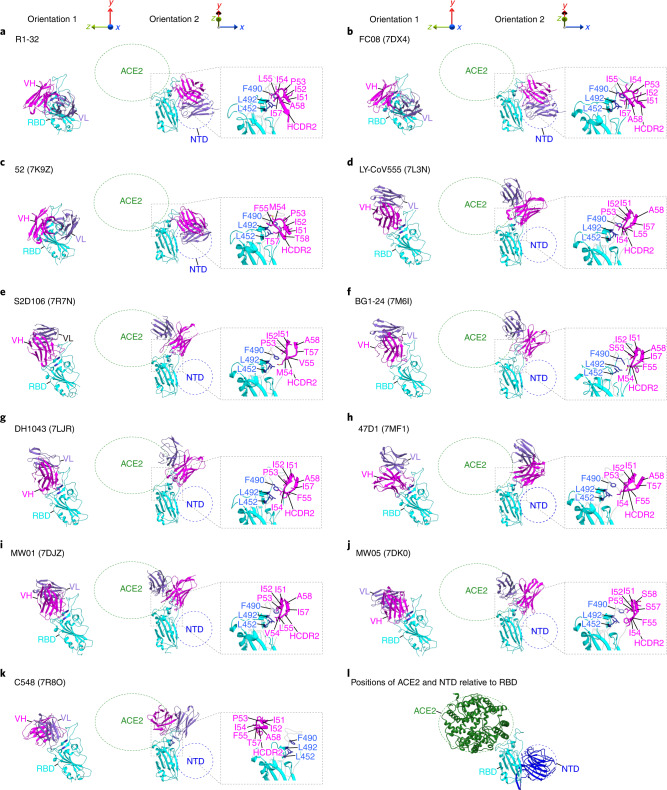
Epitopes and binding modes of R1-32, R1-32-related and class 2 VH1-69 antibodies. **a**–**c**, R1-32 (**a**), FC08 (**b**) and 52 (**c**) are antibodies of the newly defined R1-32-represented class. **d**–**k**, Reported class 2 VH1-69 antibodies. Except for **k** (C548), all shown VH1-69 antibodies interact with L452, F490 and L492 (the R1-32 HCDR2 epitope) using the hydrophobic HCDR2. Fab-VH, Fab-VL and RBD are coloured in magenta, purple and cyan, respectively. The green circles show the position of ACE2 when bound to RBD; the blue circles show the position of NTD in relation to an RBD in a ‘down’ position as illustrated in **l**. Epitopes of HCDR2 and hydrophobic residues involved in antibody-antigen interaction are shown in dashed boxes.

**Fig. 6:**
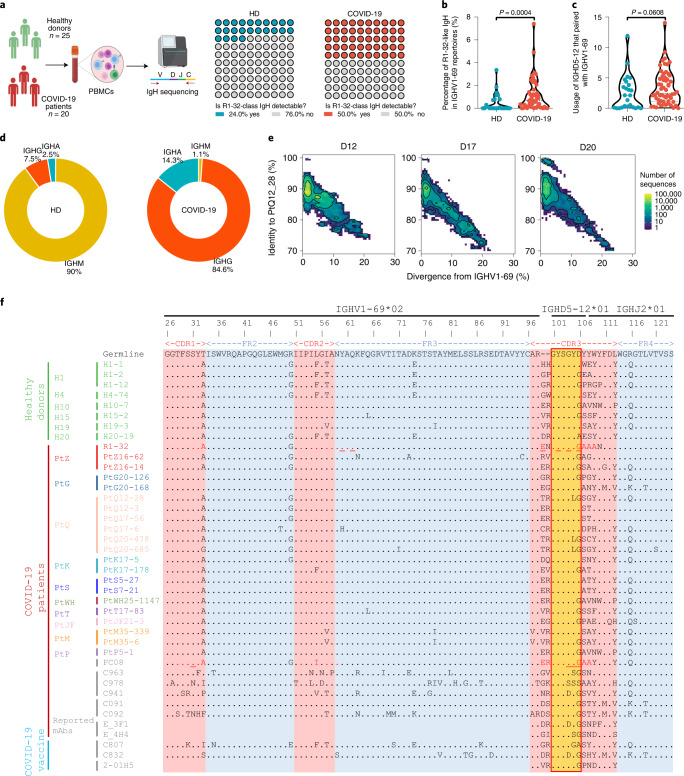
High occurrence of R1-32-like antibodies in healthy individuals (HD) and their rapid induction in COVID-19 patients. **a**, Waffle charts showing the occurrence of R1-32-like IgH sequences in a cohort of 20 COVID-19 patients and 25 healthy donors^22–24^. Created with BioRender.com. **b**, Violin plot showing the comparison of percentages of R1-32-like IgH sequences in IGHV1-69-encoded IgH gene repertoires across the COVID-19 patients and healthy donors. **c**, Violin plot showing the comparison of usage of IGHD5-12 that paired with IGHV1-69 across the COVID-19 patients and healthy donors. Statistics for the violin plots are unpaired non-parametric Mann-Whitney tests. **d**, Pie charts showing the isotype distribution of R1-32-like IgH sequences in COVID-19 patients and healthy donors. **e**, Identity-divergence plot showing the clonal expansion of R1-32-like antibody lineages from day 12 to day 20 after symptom onset in a representative COVID-19 patient. All IGHV1-69 sequences in the repertoires were plotted as a function of sequence divergence from IGHV1-69 germline gene (*x* axis) and sequence identity to an R1-32-like clone PtQ12_28 (*y* axis). Colour gradient indicates sequence density. **f**, Multiple sequence alignment of R1-32 with selected R1-32-like IgH sequences identified from the COVID-19 patients and healthy donors^22–24^, as well as several previously reported R1-32-like mAb sequences^25–29^. The germline gene sequence is used as the reference. The RBD-contact residues in R1-32 or FC08 are coloured red, with those involved in hydrogen bonding underlined. The amino acid residues identical to the germline sequence are dotted.

**Extended Data Fig. 7:**
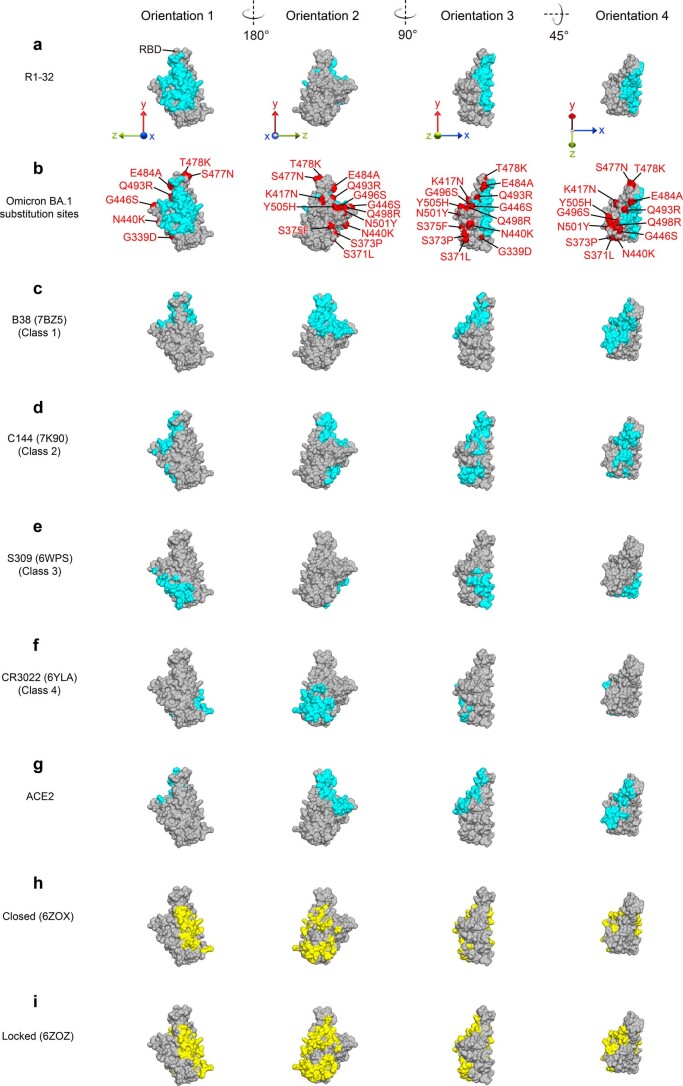
Epitopes of representative mAbs of different RBD targeting antibody classes compared to the ACE2 binding surface and buried surfaces of RBD in closed and locked RBD ‘down’ spike trimers. RBD molecular surfaces are colored in gray. Epitopes of **a**, R1-32, **c**, B38 (ref. ^59^), **d**, C144 (ref. ^2^), **e**, S309 (ref. ^60^), **f**, CR3022 (ref. ^61^), and the binding surface of **g**, ACE2 (ref. ^50^) are highlighted in cyan. **b**, Epitopes of R1-32 (cyan) and substituted residues (red) in the Omicron BA.1 variant are highlighted. RBD surface areas buried by NTD in **h**, closed and **i**, locked spike trimers^8^ are highlighted in yellow.

**Extended Data Fig. 8:**
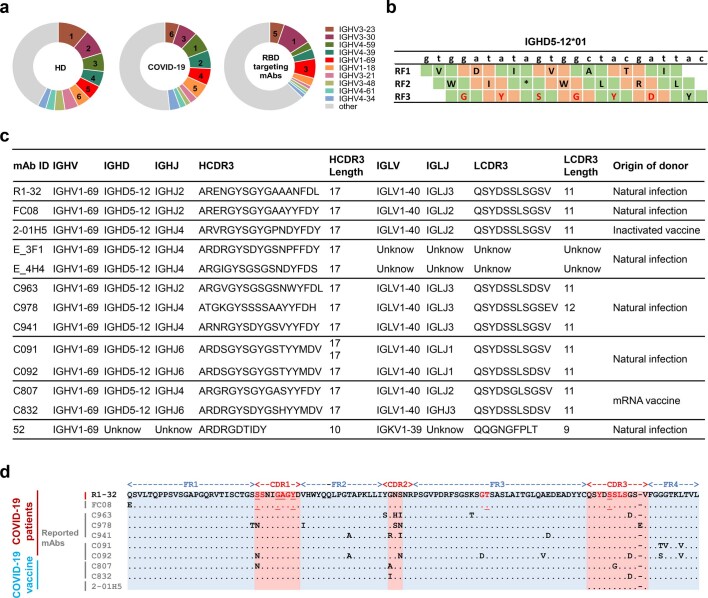
Analysis of IGHV1-69-encoded R1-32 like or R1-32 related RBD targeting antibodies. **a**, Pie charts showing the IGHV usage in healthy donors (HD, left), COVID-19 patients (middle), and published RBD targeting mAbs (right). Top 10 IGHVs are shown according to their rank in healthy donors. **b**, Three reading frames (RF1-3) of IGHD5-12 are shown. The GYSGYD motif is encoded by RF3. **c**, A summary of representative VH1-69 R1-32 like and R1-32 related mAbs reported from independent studies, except for 52, all are encoded by IGHV1-69, IGHD5-12, and IGLV1-40 and exhibit high sequence similarity to R1-32. **d**, Alignment of R1-32 like antibody light chain sequences. The RBD-contact residues in R1-32 and FC08 are colored in red with those involved in hydrogen-bonding underlined. The identical amino acid residues compared to the R1-32 light chain sequence are dotted.

**Extended Data Fig. 9:**
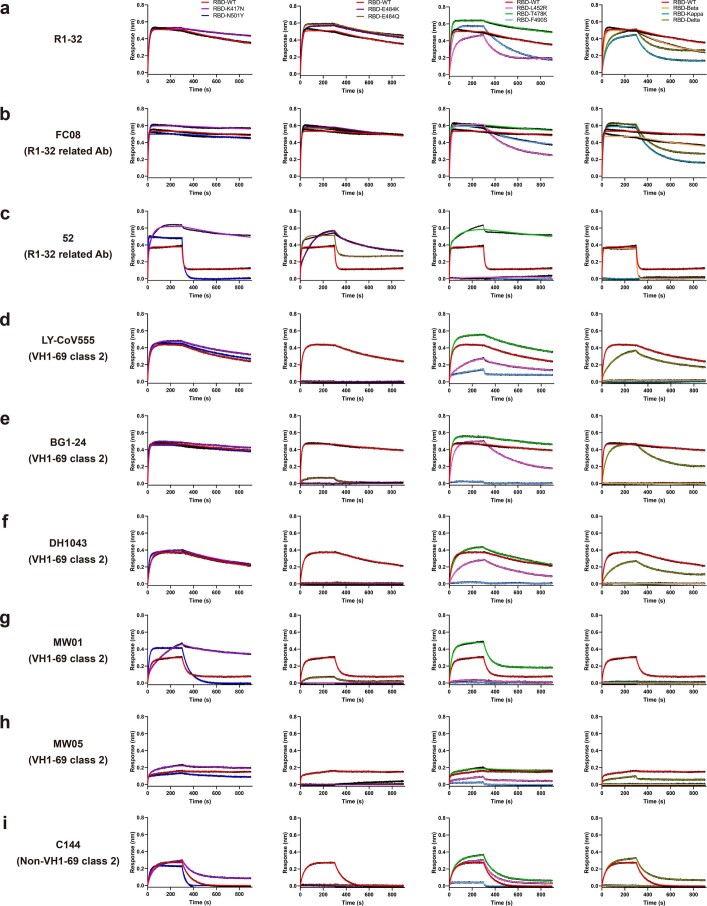
Effect of selected RBD substitutions on binding of selected antibodies. **a**, VH1-69 antibody R1-32; R1-32 related VH1-69 antibodies **b**, FC08, **c**, 52; selected class 2 VH1-69 antibodies **d**, LY-CoV555, **e**, BG1-24, **f**, DH1043, **g**, MW01, **h**, MW05; and a non-VH1-69 class 2 antibody **i**, C144 were immobilized onto Protein A biosensors. Binding to RBDs with indicated substitutions and RBDs of variants was compared to the wild-type RBD binding curves. Kinetic parameters are summarized in Supplementary Table 8.

RBD-targeting antibodies can be broadly grouped into 4 classes on the basis of epitope locations (Extended Data Fig. 7 and Supplementary Fig. 3)^2^. By analysing a set of 179 antibody-RBD complex structures in the Protein Data Bank (PDB), we found that the semi-cryptic R1-32 epitope does not belong to any of these 4 classes. Further, only 2 antibody-RBD complex structures in the PDB (52 (ref. ^9^), FC08 (ref. ^10^)) target the same epitope. It seems that this set of 3 antibodies is rare, at least among structures that have been submitted to the PDB. We note that 52 and FC08 antibodies use the VH1-69 gene, but that structural studies of both antibodies used RBD complexes and neutralization mechanisms were not studied. R1-32, FC08 and 52 bind RBD in a very similar fashion (Fig. 5a–c), although they differ in HCDR3 sequences (Fig. 6f and Extended Data Fig. 8c). By comparing RBD complex structures of R1-32 and FC08, we identified a GYSGYG/D motif that is shared among HCDR3 loops of R1-32 and FC08, and is probably responsible for higher-affinity binding. Backbone carbonyl oxygens and amide nitrogens in this motif mediate extensive hydrogen bonding with the HCDR3 epitope (Figs. 2c (right) and 6f). This motif is absent from 52, which features a shorter HCDR3 loop and binds more weakly to RBD with very fast dissociation in our BLI assays (Extended Data Fig. 9c), suggesting that a shorter HCDR3 loop in combination with the hydrophobic VH1-69 HCDR2 is strong enough to enable an R1-32-like binding mode.

We analysed 13 available VH1-69 antibody-RBD complex structures in the PDB, and identified 1 class 1 antibody (CV503 (ref. ^11^)), 8 class 2 antibodies (DH1043 (ref. ^12^), LY-CoV555 (ref. ^13^), 47D1 (ref. ^14^), BG1-24 (ref. ^15^), MW01 (ref. ^16^), MW05 (ref. ^16^), S2D106 (ref. ^17^), C548 (ref. ^18^)), 1 class 3 antibody (FD20 (ref. ^19^)), 1 class 4 antibody (S2X259 (ref. ^20^)) and the 2 R1-32-related antibodies (52, FC08) (Supplementary Fig. 3). Although the 3 VH1-69 antibodies in classes 1, 3 and 4 (CV503, FD20, S2X259) bind far away from the R1-32 epitope, remarkably, 9 VH1-69 antibodies (excluding C548) use hydrophobic HCDR2 loops to contact the HCDR2 epitope (L452, F490 and L492) (Fig. 5b–j). We also found that class 2 VH1-69 antibodies are incompatible with the binding mode of R1-32 due to clashes between HCDR3 loops and RBD. Consequently, these antibodies feature a ~120° rotation in binding orientation such that their light chains are orientated to clash with ACE2 binding and their epitopes are fully exposed (Fig. 5d–k). These analyses reveal that the hydrophobic HCDR2 epitope can serve as an anchor point to support two different binding modes of VH1-69 antibodies. The strong HCDR2 anchor point, being central in the exposed part of the unique semi-cryptic epitope, conferred R1-32 with the ability to alter spike conformations, resulting in spike destruction. This neutralization mechanism differentiates R1-32 from class 2 and class 3 antibodies that target nearby fully exposed epitopes and are unable to alter spike structure. Therefore, we propose that R1-32, and the related FC08 and 52 antibodies define a distinct class of RBD-targeting antibodies.

### R1-32-like antibodies are evaded by RBD substitutions at position 452

The Delta variant (L452R/T478K in RBD) emerged by outcompeting Kappa and B.1.617.3 (both with L452R/E484Q in RBD) variants with E484Q substitutions in India and subsequently displaced the Alpha, Beta and Gamma variants (with substitutions at 417, 484 and 501) worldwide. Point mutation data confirmed that K417N, E484K and N501Y have no effect on R1-32, FC08 and 52 binding (Extended Data Fig. 9a–c), consistent with the fact that the side chain of residue 484 faces away from the R1-32 binding surface and residues 417 and 501 are outside of the R1-32 epitope (Fig. 2e and Supplementary Fig. 1). We also found that T478K in the Delta variant has no effect on binding of tested antibodies (Extended Data Fig. 9). L452R greatly reduced RBD binding by R1-32 and FC08 (Extended Data Fig. 9a,b), and in line with a previous study^9^, completely abolished binding by 52 (Extended Data Fig. 9c). L452R has no effect on binding of non-VH1-69 class 2 antibody C144 (Extended Data Fig. 9i). Although L452R affected RBD binding by class 2 VH1-69 antibodies to a certain extent (Extended Data Figs. 6d–h and 9d–h), E484K is much more efficient in completely abolishing binding of both VH1-69 and non-VH1-69 class 2 antibodies (Extended Data Fig. 9d–i).

On the basis of these observations, we hypothesize that substitutions at 452 in the HCDR2 epitope may evade R1-32-like antibodies rather than class 2 antibodies (although we note that F490S is efficient in evading both class 2 and R1-32-related antibodies; Extended Data Fig. 9).

The first emerged Omicron BA.1 variant, which renders most known RBD-targeting antibodies ineffective^21^, does not contain any substitutions at 452 and 490 (Fig. 2e). The spike substitutions it carries are either peripheral to (for example 484, 493) or outside of the R1-32 epitope (Fig. 2e, Extended Data Fig. 7b and Supplementary Fig. 1). Consistent with this, we found that R1-32 maintains high-affinity binding to the Omicron BA.1 RBD/spike trimer (Fig. 1a,b) and neutralizes Omicron BA.1 pseudovirus (Fig. 1c).

### A population antibody response comprises R1-32-like antibodies

**Extended Data Fig. 10:**
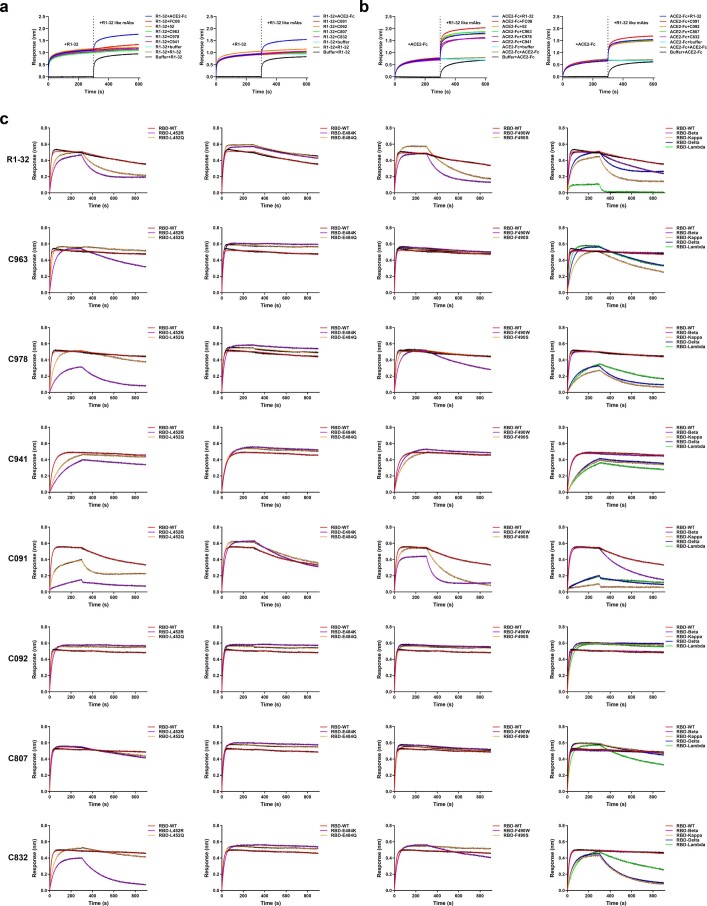
Characterization of R1-32 like mAbs. **a**, Competition between R1-32 like mAbs and R1-32 for binding to SARS-CoV-2 RBD. **b**, Competition between R1-32 like mAbs and ACE2-Fc for binding to SARS-CoV-2 RBD. Competition assays were performed by BLI. SARS-CoV-2 RBD was immobilized onto Anti-His biosensors, immobilized RBD was saturated with R1-32 or ACE2-Fc, followed by incubation with R1-32 like mAbs (colored lines). As controls, biosensors immobilized with RBD was first equilibrated in buffer before submerging into ACE2-Fc or R1-32 solutions (black traces). **c**, Binding of R1-32 like mAbs to RBD point mutants or VOC RBDs. R1-32 and R1-32 like mAbs (C963, C978, C941, C091, C092, C807, and C832) were immobilized onto Protein A biosensors and submerged into RBD solutions (at 200 nM) to record sensorgram traces. Kinetic parameters are summarized in Supplementary Table 9.

The hypothesis that recurrent substitutions at 452 are related to evasion of R1-32-like antibodies suggests that R1-32-like antibodies may be common in the population. We searched immunoglobulin heavy chain (IgH) repertoires of 20 COVID-19 patients and 25 healthy donors we published previously^22–24^ for VH1-69 transcripts having at least 80% amino acid homology to either R1-32 or FC08 in HCDR3, or for a GYSGYG/D motif in HCDR3 (see Methods for detailed selection criteria) (Fig. 6f). R1-32-like IgH sequences were detected in 50% (10/20) of COVID-19 patients and 24% (6/25) of healthy donors (Fig. 6a). These sequences are germline-like, explaining their high occurrence (Fig. 6f and Extended Data Fig. 8b). The percentage of R1-32-like IgH sequences in IGHV1-69-encoded repertoires of COVID-19 patients was significantly higher than that present in healthy donors (Fig. 6b), suggesting a noticeable clonal expansion of this lineage of antibodies after SARS-CoV-2 exposure. This is accompanied by a slight increase in IGHD5-12 usage that rearranges with IGHV1-69 in COVID-19 patients (Fig. 6c). We observed the clonal expansion of R1-32-like IgH sequences in a representative COVID-19 patient by tracking the longitudinal samples collected at days 12 to 20 post symptom onset (Fig. 6e). We noted that R1-32-like IgH sequences that present in SARS-CoV-2-exposed antibody repertoires are mainly switched to IgG (encoded by IGHG, 84.6%) or IgA (encoded by IGHA, 14.3%) isotypes. However, 90% of R1-32-like IgH sequences detected in SARS-CoV-2-unexposed antibody repertoires are IgM (encoded by IGHM) isotype that is usually expressed in naïve B cells (Fig. 6d). This result indicates that naïve B-cell-expressing R1-32-like antibodies had rapidly undergone class switching after SARS-CoV-2 infection. In addition, by sequence similarity, we identified several structurally uncharacterized R1-32-like mAbs including 2-01H5 (ref. ^25^), E_3F1 and E_4H4 (ref. ^26^), C963, C978 and C941 (ref. ^27^), C091 and C092 (ref. ^28^), C807 and C832 (ref. ^29^) isolated from different COVID-19 convalescents or vaccinees in 5 previous independent studies (Fig. 6f and Extended Data Fig. 8c). We tested the above identified R1-32-like mAbs with complete IgH/IgL sequences (C963, C978, C941, C091, C092, C807 and C832), these mAbs being isolated using single-cell PCR. We found that all of them competed with R1-32 for RBD binding (Extended Data Fig. 10a) and none of them competed with ACE2 binding (Extended Data Fig. 10b). Similar to R1-32, substitutions at 452 and 490 generally weaken binding by R1-32-like mAbs, and 484 substitutions have no effect on R1-32-like mAb binding (Extended Data Fig. 10c). Among these R1-32-like mAbs, C092 was resistant to substitutions at 452 and 490 (Extended Data Fig. 10c), suggesting that a subset of R1-32-like mAbs may overcome escape by variants with 452 and 490 changes. Although R1-32 and FC08 were discovered using phage display, their heavy and light chain compositions are very similar to those of naturally paired R1-32-like mAbs isolated using single-cell PCR: all of these mAbs use IGHD5-12, IGLV1-40, 17-residue HCDR3 and almost identical LCDR3 loops except for C978 (Fig. 6f and Extended Data Fig. 8c,d).

We propose that this is evidence for a convergent shared antibody response in the broad population, induced by SARS-CoV-2 spike antigen. Collectively, we found that R1-32-like antibodies were commonly induced after SARS-CoV-2 infection or vaccination, and we propose that these antibodies should be classified as a separate lineage of shared antibodies, in addition to being a distinct class of RBD-targeting antibodies.

## Discussion

It has been proposed that substitutions in spike proteins of dominant SARS-CoV-2 variants enable evasion of population immunity^1^. L452R is inefficient in evading class 2 and 3 antibodies including C144, REGN10987 (ref. ^30^) and S309 (ref. ^31^), while class 1 and 4 antibodies bind far away from 452. Although it has been proposed that L452R may improve other aspects of spike function, thereby increasing virus infectivity^32,33^, we identified that recurrent substitutions at, or around position 452, affect binding of commonly induced VH1-69 antibodies, in particular R1-32-like antibodies.

Our findings partly explain why 452 and 490 are recurrent substitution sites in the RBD. Omicron BA.1 variant emerged with 484 and 417/501 substitutions but curiously lacked substitutions at 452 or 490, and was therefore sensitive to R1-32 neutralization. A recent study reported that memory B cells expressing non-ACE2-competing IGHV1-69-IGHD5-12/IGLV1-40 antibodies were recalled in Omicron BA.1 (with L452) breakthrough infections^34^. F490S was first identified in the Lambda variant, and again in certain Delta subvariants^35^. F490R/S has been identified in B.1.640.1 and B.1.640.2 variants^36^. Of note, L452Q, L452M and L452R are associated with the emergence of Omicron BA2.12.1, BA2.13, BA.4 and BA.5 subvariants^37^. These Omicron subvariants have been shown to cause large-scale escapes of IGHV1-69-IGHD5-12/IGLV1-40 antibodies such as FC08, BD-380, BD-421, BD-901, C091 and XGv-271 through L452 substitutions^38^. Emergence of these subvariants suggests a continued immune pressure on the identified VH1-69 ‘HCDR2’ epitope.

It is not clear why R1-32-like antibodies impose a strong immune pressure over SARS-CoV-2 virus. Our BLI data showed that SARS-CoV-2 spike can use conformational masking to impair binding of diverse RBD-targeting antibodies. Through HCDR2, R1-32-like and R1-32-related antibodies can bind all known spike conformations, particularly the RBD ‘down’ conformations, and in so doing expose RBD, or disrupt spike structures (Fig. 3 and Extended Data Fig. 6). We hypothesize that binding of this class of antibodies impairs spike protein conformational masking, resulting in increased antibody binding and elevated immune pressure.

VH1-69 is one of the most frequently used genes in the human antibody repertoire (Extended Data Fig. 8a). Its hydrophobic HCDR2 loop is a unique feature of VH1-69 antibodies, and this feature has been implicated in antibody responses to diverse pathogens, including influenza, HIV, hepatitis C viruses and more^7^.

Here we identify an important role for VH1-69 HCDR2 in anti-SARS-CoV-2 immunity, which probabely contributes to the genesis of recurrent substitutions at 452 and 490. We conclude that immune pressures exerted by the population antibody response to SARS-CoV-2 is likely to underpin evolution of substitutions at positions 452 and 490. These mutation ‘hot spots’ should be continuously monitored and future studies should address the potential pathogenic consequences of VH1-69 antibody evasion by SARS-CoV-2.

## Methods

### Convalescent patients

A total of 6 confirmed COVID-19 patients cared by Guangzhou Eighth People’s Hospital, China, from 21 January to 19 February 2020 were enroled in this study. Whole blood was collected from the COVID-19 patients during hospitalization (more patient information is given in Extended Data Fig. 1a and Supplementary Table 4). We obtained informed consent from all enroled patients. This study was approved by the Ethics Committee of Guangzhou Eighth People’s Hospital (REC ref: AF/SC-02/01.6).

### Cells and viruses

Expi293F cells (Thermo Fisher, A14527) were maintained in Expi293F culture medium (Thermo Fisher, A1435101) and incubated at 37 °C in an orbital incubator shaker with humidified atmosphere containing 5% CO_2_. Cells (293T, ATCC, CRL-3216; and Vero E6, ATCC, CRL-1586) were cultured at 37 °C in Dulbecco’s modified Eagle medium (DMEM) containing 10% fetal bovine serum (FBS).

Wild-type authentic SARS-CoV-2 viruses: SARS-CoV-2/human/CHN/IQTC01/2020 used for animal experiment was isolated from COVID-19 patients in Guangzhou (GenBank accession no. MT123290)^39^; 2019-nCoV BetaCoV/Wuhan/WIV04/2019 (GenBank accession no. MN996528) is stored at Microorganisms and Viruses Culture Collection Center, Wuhan Institute of Virology, Chinese Academy of Sciences (CSTR: 1633.06.IVCAS 6.7512)^40^; SARS-CoV-2 Beta variant (CSTR: 1633.06.IVCAS.6.7552) and Delta variant (CSTR.16698.06.NPRC 6.CCPM-B-V-049-2105-8) are stored at the National Pathogen Resource Center (NPRC).

### hACE2 transgenic mice

The human ACE2 transgenic mice (C001191) were provided by Cyagen Biosciences. The mice were kept in Biosafety Level-2 housing and given access to standard pellet feed and water following the standard operational procedures (SOPs) of Guangzhou Institutes of Biomedicine and Health. The viral challenge experiments were then conducted in a Biosafety Level-3 animal facility strictly following SOPs.

### Construction of an single-chain variable fragment (scFv) phage display libraries

Peripheral blood mononuclear cells (PBMCs) were isolated with Opti-Prep lymphocyte separation solution (Axis Shield PoC AS) following the manufacturer’s instructions. Total RNA was extracted from PBMCs of convalescent SARS-CoV-2 patients using TRIzol (Invitrogen, 15596018) according to the manufacture’s instruction. Both total RNA and mRNA were reversely transcribed into complementary DNA by iScript cDNA synthesis kit (Bio-Rad, 1708891). Next, variable regions of antibody heavy chain (VH) and light chain (VL) were amplified using human antibody-specific primers. Then, scFvs that comprise a single polypeptide with VH and VL domains linked together by a flexible glycine-serine linker were generated. The scFvs were assembled into the pCANTab5E phage display vector using the T4 DNA ligase kit (Takara, 2011B). Finally, the assembled products were transformed into TG1 *E. coli* cells (Lucigen, 60502-2) and resulted in 6 libraries, one for each of the 6 patients. Each library contained a barcode and had a diversity of approximately 4 × 10^8^ clones.

### Screening the scFv phage libraries

The libraries were panned against the purified recombinant SARS-CoV-2 RBD protein (Sino Biological, 40592-V08B). Usually 1 µg ml^−1^ antigen was coated into immunotubes overnight at 4 °C. The next day, tubes were washed 3 times with phosphate-buffered saline (PBS), then filled with PBS-5% skimmed milk to block at 37 °C for 2 h. After standard washes, 10^10^ purified phages in 2 ml of PBS-5% skimmed milk were added into tubes and incubated at room temperature for 2 h. Supernatant was removed and tubes were washed 10 times with PBS-0.1% Tween-20. The remaining phage was eluted by adding 500 μl of 1 mg ml^−1^ trypsin-PBS and rotating for 10 min at room temperature. The eluted phages were amplified by infecting TG1 *E. coli* cell cultures. At round 2, tubes were coated with the same antigen as round 1, and 10^6^ purified phages from round 1 (approximately 10^4^ clones) were added. To enrich phages for specific binding, tubes were washed 20 times with PBS-0.1% Tween-20 at round 2. Polyclonal phage ELISA was carried out at round 2 and when phages were found to be sufficiently enriched, single colonies were picked for monoclonal phage ELISA.

### Polyclonal and monoclonal phage ELISAs

ELISA plates (96-well) were coated overnight at 4 °C with 100 µl per well of purified recombinant SARS-CoV-2 RBD at 0.5 µg ml^−1^ in PBS. After washing with PBS-0.1% Tween-20, the plates were blocked using PBS-5% skimmed milk and incubated for 2 h at 37 °C. For polyclonal phage ELISA, phage library or amplified eluted phages were serial diluted in PBS-5% skimmed milk starting from 1:2 before 100 µl were added to wells. Phage solution was discarded after 1 h incubation at room temperature and wells were washed with PBS-0.1% Tween-20. Horseradish peroxidase (HRP)-anti-M13 (NbBiolab, S004H) was added at a dilution of 1:5,000 in PBS-5% skimmed milk before incubation at room temperature for 1 h. After washing with PBS-0.1% Tween-20 6 times, 3,3′,5,5′-tetramethylbenzidine (TMB) solution (Merck Millipore, ES001) was used as the substrate, and absorbance at 450 nm was measured in a microplate reader.

For monoclonal phage ELISA, a single colony was picked from plates amplifying eluted phages and cultured in 96-deep-well plates containing 200 µl per well of medium and grown shaking (250 r.p.m.) at 37 °C. When the optical density (OD)_600_ reached 0.5, to each well of the single-colony culture, 10^9^ M13K07 helper phages (25 µl per well) (NEB, N0315S) were added before growing with shaking (250 r.p.m.) overnight at 30 °C. The supernatant of the single-colony culture was used for monoclonal ELISA with a 1:1 dilution with PBS-5% skimmed milk following the same procedures as polyclonal phage ELISA. Briefly, the supernatants of single-colony cultures were incubated in 96-well ELISA plates for 1 h before plates were washed. Then plates were incubated with HRP-anti-M13. After standard washing procedures, TMB solution was used as the substrate. Finally, absorbance at 450 nm was measured in a microplate reader. Strong single binders were sequenced and analysed using the International Immunogenetics information system (IMGT) (http://imgt.org). Extended Data Fig. 1b describing antibody isolation was generated with BioRender.com.

### Human monoclonal antibody expression and purification

The antibody VH and VL region genes were amplified and cloned into the expression vectors pCMV3-IgG1, pCMV3-Lambda and pCMV3-Kappa using Clone Express II one-step cloning kit (Vazyme, C112). The plasmids of paired IgH and IgL genes were transiently co-transfected into the Expi293F expression system at a ratio of 1:1 (IgH:IgL) using EZ Cell transfection reagent (Life-iLab Biotech, AC04L092). Recombinant mAbs were produced following the manufacturer’s protocol. Briefly, antibodies in the cell culture supernatants were collected at 5 d post transfection before purification using MabSelect agarose (Cytiva) according to the manufacturer’s instructions. The purified mAbs were concentrated and buffer-exchanged into PBS by a 50 kDa MWCO Amicon Ultra filtration device (Merck Millipore).

### Fab fragment production

To obtain the R1-32 Fab, the R1-32 VH and the human IgG1 constant region CH1 were amplified from the IgH gene by primers: (F: 5′-TGGCTACCAGGGTGCTGAGCGAAGTGCAGCTGGTGCA-3′; R: 5′-CGAATTCGGCGGCCGCTTAGTCACAAGATTTGGGCTCAAC-3′). The plasmids of paired Fab-IgH and IgL were transiently co-transfected into the Expi293F cells at a ratio of 1:1 (Fab-IgH:IgL) using EZ Cell transfection reagent according to the manufacturer’s instructions. The supernatant was collected at 5 d post transfection. The Fab was purified with LambdaFabSelect affinity chromatography (Cytiva). The Fab bound to the resin surface was eluted with 0.1 M glycine (pH 2.5) into 1/4th volume 1 M Tris-HCl (pH 8.5) and concentrated by a 30 kDa MWCO Amicon Ultra filtration device (Merck Millipore). Finally, the Fab was further purified and buffer-exchanged into PBS using a Superdex 200 increase 10/300 GL column (Cytiva).

### Determination of antibody binding activity to SARS-CoV-2 RBD by ELISA

To assess antibody binding properties, 96-well ELISA plates were coated with SARS-CoV-2 RBD at 1 µg ml^−1^ in PBS at 4 °C overnight. After standard washing and blocking procedures, 100 µl antibodies in semilogarithmic serial dilutions were added and incubated at 37 °C for 2 h. After washing plates 3 times with PBS-0.05% Tween-20, plates were incubated with 1:5,000-diluted HRP-labelled goat anti-human IgG (H+L) (Beyotime, A0201) in PBS-5% skimmed milk at 37 °C for 1 h. Plates were washed with PBS-0.05% Tween-20 6 times, TMB solution was used as the substrate, and absorbance at 450 nm was measured in a microplate reader.

### Protein expression and purification

S-GSAS/6P was constructed according to a previous report^41^; all other SARS-CoV-2 S and ACE2-Fc constructs have been previously described and were expressed and purified following the previously established protocols^8,42,43^. To obtain ACE2 without tag (referred to as ACE2), the Fc tag was removed by trypsin digestion in a reaction mixture containing 5 mg ACE2-Fc and 50 µg trypsin in PBS. After incubation for 2 h at room temperature, the digestion reaction was stopped by the addtion of 1 mM phenylmethylsulfonyl fluoride (PMSF), before the mixture was reloaded onto a Protein A column (Cytiva) to remove the undigested ACE2-Fc and Fc tag. The ACE2 was further purified by a Superdex 200 increase 10/300 GL column (Cytiva) in PBS before concentrating and storage at −80 °C.

The coding sequence of SARS-CoV-2 RBD (residues 319–541) with an N-terminal mu-phosphatase signal peptide and a C-terminal 6-His-tag was cloned into a pCDNA3.1 vector. The expression vector was transiently transfected into Expi293F cells using polyethylenimine. At 5 d after transfection, the supernatant of the cell culture was collected and added with 25 mM phosphate pH 8.0, 300 mM NaCl, 5 mM imidazole and 0.5 mM PMSF, and recirculated onto a HiTrap TALON crude column (Cytiva) 3 times. Subsequently, the column was washed with 100 ml of buffer A (25 mM phosphate pH 8.0, 5 mM imidazole, 300 mM NaCl), and protein was eluted with a 100 ml linear gradient to 100% buffer B (25 mM phosphate pH 8.0, 300 mM NaCl, 500 mM imidazole). Fractions containing the RBD were pooled, concentrated with a 10 kDa MWCO Amicon Ultra filtration device (Merck Millipore) and buffer-exchanged into PBS. Concentrated RBD was aliquoted, flash frozen in liquid nitrogen and stored at −80 °C. All SARS-CoV-2 RBD variants were purified as described above.

### Binding kinetics and affinity assessment of mAbs by BLI

Binding kinetics and affinities of mAbs against SARS-CoV-2 spikes or RBDs were assessed by BLI on an Octet RED96 instrument (Sartorius). All steps were performed at 25 °C at an orbital shaking speed of 1,000 r.p.m. All reagents were formulated in PBS-TB buffer (PBS with 0.02% v/v Tween-20 and 0.1% w/v BSA). Before the experiments, all biosensors were pre-equilibrated in the PBS-TB buffer for 10 min. mAbs (11 µg ml^−1^) were immobilized onto Protein A biosensors (Sartorius) to a level of ~1.7 nm. After a 60 s baseline step in PBS-TB, the mAb-loaded biosensors were exposed (300 s) to the analytes (spikes or RBDs) (from 200 nM to 3.125 nM in 2-fold serial dilutions) to measure association before the sensors were dipped into PBS-TB (600 s) to measure dissociation of analytes from the biosensor surface. Data for which responses in the association phases were >0.1 nm were aligned, reference-subtracted (to blank sensors), inter step corrected (to the association step) and further analysed using the FortéBio data analysis software HT v12.0.2.59 (Sartorius) by fitting to single or double phase association and dissociation kinetics to determine *k*_on_, *k*_off_ and *K*_D_ (the binding constant determined from the ratio of the individual rate constants) as previously described^43^. Raw data and fits were plotted in GraphPad Prism 8.0.

Affinities of mAbs with SARS-CoV-2 mutant RBDs were evaluated by immobilizing the antibody mAbs (11 µg ml^−1^) on Protein A biosensors to a level of ~1.7 nm. After a 60 s baseline step in PBS-TB, the biosensors were exposed (300 s) to the mutant RBDs (200 nM) and then dipped (600 s) into PBS-TB to measure dissociation of the antigen from the biosensor surface. Data were analysed using the FortéBio data analysis software HT.

### Antibody competition assay by BLI

The competitive binding between antibodies or between antibodies and ACE2 were measured using a Gator label-free bioanalysis system (GatorBio). Anti-His biosensors (GatorBio) were pre-equilibrated in PBS-0.02% Tween-20 buffer before being immobilized with 2 μg ml^−1^ recombinant SARS-CoV-2 RBD-His protein (Sino Biological, 40592-V08B). Biosensors were saturated with 10 μg ml^−1^ of the first ligand (antibody or ACE2). Finally, binding of the second ligand (or buffer as control) was measured for 300 s. Data were analysed by the Gator data analysis software (GatorBio).

### Pseudovirus neutralization assay

Pseudotyped lentiviruses were produced in 293T cells as previously described^44^ by co-transfecting a plasmid expressing S protein, a packaging vector and a reporter vector carrying an expression cassette of firefly luciferase. The 3× serially diluted antibodies were incubated with the SARS-CoV-2 pseudotyped virus at 37 °C for 1 h. The mixture was subsequently incubated with 293T-ACE2 cells for 72 h. The cells were washed twice with PBS and lysed with lysis buffer before measuring luciferase activity. The neutralization titre was calculated as the antibody dilution at which the luciferase activity was reduced to 50% of that from the virus-only wells.

### Authentic virus neutralization assay

Vero E6 cells were seeded in 24-well plates (2 × 10^5^ cells per well) 1 d before infection. Antibodies were diluted in DMEM medium at indicated concentrations and incubated with wild-type SARS-CoV-2 viruses (2019-nCoV BetaCoV/Wuhan/WIV04/2019; GenBank accession no. MN996528), SARS-CoV-2 Beta variant (CSTR: 1633.06.IVCAS.6.7552) or SARS-CoV-2 Delta variant (CSTR.16698.06.NPRC 6.CCPM-B-V-049-2105-8) (50 TCID_50_) at 37 °C for 1 h or 4 °C for 16 h. The cells were incubated with 100 μl of the antibody-virus mixture for 1 h, covered with a layer of 2% methyl cellulose and incubated at 37 °C with 5% CO_2_ for 4 d. The cells were fixed with 3.7% formaldehyde for 24 h and stained with 1% crystal violet to visualize plaques. Experiments were performed in triplicates. Percentage neutralization was calculated by normalization to plaque numbers in parallel virus-only wells.

### Animal experiments

In vivo protection efficacy of the R1-32 antibody was evaluated using a hACE2 transgenic mouse model^6^. The animal study protocol was approved by the Ethics Committee of Guangzhou Institutes of Biomedicine and Health, Chinese Academy of Sciences (IACUC: 2020025). All work with live SARS-CoV-2 was conducted in Biosafety Level-3 Laboratories. Female mice (20 weeks old) were randomly divided into three groups (5 mice per group), including two therapeutic groups and one control group. Mice were inoculated intranasally with 5 × 10^5^ p.f.u. wild-type SARS-CoV-2/human/CHN/IQTC01/2020 (GenBank accession no. MT123290)^39^. For the therapeutic groups, each mouse received a single intraperitoneal injection of either 20 mg kg^−1^ high dose or 4 mg kg^−1^ low dose of R1-32 at 1 h after infection, and the control group was injected with an equivalent volume of PBS. Body weights were monitored and recorded for 4 d. All mice were euthanized at 3 d post infection (dpi), and the lungs were collected for viral load analysis and hematoxylin and eosin (H&E) staining. Figure 1d describing animal experiments was generated with BioRender.com.

Viral RNA quantification was performed by RT-qPCR using the QuantiTect SYBR Green RT-PCR kit (Qiagen, 204243). Lung homogenates were prepared by homogenizing perfused right lungs using an electric homogenizer. The supernatant was collected to extract total RNA using TRIzol according to the manufacture’s instruction. Primers targeting the SARS-CoV-2 S gene were used for RT-qPCR: qF (5’- CAATGGTTTAACAGGCACAGG–3’), qR (5’- CTCAAGTGTCTGTGGATCACG-3’). The left lungs were fixed in 4% (v/v) paraformaldehyde solution. Tissue paraffin sections (2–4 µm) were stained with H&E. The slices were examined and imaged by light microscopy.

### Negative-stain electron microscopy

SARS-CoV-2 S-R spike at 1 mg ml^−1^ (7.09 µM) was incubated in a 1:2.2 molar ratio with ACE2-Fc, IgGs (B38, rmAb23 or R1-32) or in a 1:1.1 molar ratio with R1-32 Fab for 1 h or 24 h at room temperature. An aliquot of 3 µl diluted sample (maintaining a spike protein concentration of 0.03 mg ml^−1^) was absorbed to freshly glow-discharged (15 mA, 45 s) carbon-coated copper grids for 30 s, followed by blotting away excess liquid with filter paper. The grids were washed twice with water and stained twice with 0.75% (w/v) uranyl formate. Micrographs were collected using the FEI TEM Imaging and Analysis (TIA) software (FEI) on a 120 keV FEI Tecnai G2 Spirit with a 4k × 4k FEI Eagle CCD camera at a ×49,000 nominal magnification.

### Ligand-induced conformational change assay

SARS-CoV-2 S-R spike at 1 mg ml^−1^ (7.09 µM) was incubated with ACE2-Fc or antibodies at a 1:2.2 molar ratio (for R1-32 Fab, the molar ratio was 1:1.1) for 1 h at room temperature. The samples were subsequently treated with 50 μg ml^−1^ proteinase K for 30 min at 4 °C. Non-reducing SDS–PAGE loading buffer (4×) was added to each sample immediately before boiling at 98 °C for 5 min to stop the reaction. Samples were separated by SDS–PAGE and transferred onto a PVDF membrane (Millipore). After blocking with 5% (w/v) skimmed milk in TBST buffer, the membrane was incubated with a rabbit anti-SARS-CoV-2 S2 polyclonal antibody (1:2,500 dilution, Sino Biological, 40590-T62) and subsequently with a goat anti-rabbit IgG conjugated to horseradish peroxidase (1:1,000 dilution, Beyotime, A0208). The S proteins on the membrane were detected by chemical luminescence using Pierce ECL western blotting substrate (Thermo Fisher, 32106). To further visualize the effect of non-ACE2 competing antibodies on S-R spike conformation, the S-R spike protein was pre-incubated with antibodies (1 h) and before further incubation with ACE2-Fc (1 h). Samples were analysed by western blotting as described above.

### Cryo-EM sample preparation and data collection

For the S-GSAS/6P:R1-32 Fab complex, S-GSAS/6P at 5.8 mg ml^−1^ was mixed with the R1-32 Fab at a 3:1 or 3:3 molar ratio for 1 min. For the S-GSAS/6P:R1-32 Fab:ACE2 complex, the S-GSAS/6P was mixed with R1-32 Fab and ACE2 at a 3:3:3 molar ratio for 1 min. The mixture (3 µl) was supplemented with 0.1% octyl-glucoside (Sigma-Aldrich, V900365) immediately before being applied to glow-discharged (15 mA, 30 s) holey carbon grids (Quantifoil, Cu R1.2/R1.3). The grids were blotted for 2 s with a force of 4, and then plunge-frozen into liquid ethane using a Vitrobot (Thermo Fisher) at 4 °C and 100% humidity. Cryo-grids were loaded into a Talos Arctica electron microscope (Thermo Fisher) operating at 200 keV for data collection using the Serial EM software^45^. Micrographs were recorded at a nominal magnification of ×45,000 on a K3 direct detection camera (Gatan) with a range of defocus between −0.8 and −2.5 μm. Each movie was collected with a dose rate of 30 e^−^ per pixel per second, fractioned into 27 frames and exposed for 1.6 s, resulting in a total dose of 63 e^−^ Å^−2^ with a calibrated pixel size of 0.88 Å.

### Cryo-EM data processing

Movies were aligned using MotionCor2 in RELION 3.1 (refs. ^46–48^), and contrast transfer function (CTF)-estimation and template-free particle picking were performed in Warp^49^. For the 3:1 S-GSAS/6P:R1-32 Fab data, 619,803 particles from 6,989 micrographs were picked, 400,197 particles from 3,346 micrographs for the 3:3 S-GSAS/6P:R1-32 Fab data, and 120,0393 particles from 13,009 micrographs for the 3:3:3 S-GSAS/6P:R1-32 Fab:ACE2 data. SARS-CoV-2 S structure in closed form (EMD-11333)^8^ was filtered to 60 Å resolution as the initial reference in the first three-dimensional (3D) classifications of each dataset. Two rounds of 3D classification were performed and resulting 3D classes in different conformations were separated. In both the 3:1 S-GSAS/6P:R1-32 Fab and the 3:3 S-GSAS/6P:R1-32 Fab data, 3:2 structures were observed in the second 3D classifications. In both datasets, 3:2 structures were combined for further processing. Auto-refinement, CTF refinement and Bayesian polishing were performed iteratively on all classified datasets. Map resolutions were estimated at the 0.143 criterion of the phase-randomization-corrected Fourier shell correlation (FSC) curve calculated between two independently refined half-maps multiplied by a soft-edged solvent mask. Final reconstructions were sharpened and locally filtered in RELION (Extended Data Figs. 3 and 4). The estimated B-factors of maps are listed in Supplementary Table 2.

All structures show flexibility in the Fab-bound RBD areas. To improve resolutions in those areas, a focused refinement was carried out for the 3:3:3 S-GSAS/6P:R1-32 Fab:ACE2 data. The 3:3:3 S-GSAS/6P:R1-32 Fab:ACE2 data were refined in C3 symmetry, and 203,698 particles in this dataset were symmetry expanded in RELION to obtain a total of 611,094 particles. The expanded particles were reconstructed by particle subtraction with a focused mask around the RBD:R1-32 Fab:ACE2 region. Final 3D auto-refinement was performed for the subtracted particles to obtain a 3.98 Å-resolution focused map, with a local resolution of 3.8 Å at its centre (Extended Data Fig. 4g).

### Model building and analysis

A structure of SARS-CoV-2 RBD:ACE2 complex (PDB: 6M0J)^50^ was fitted into the focused refined map and used as the starting model. Structures of R1-32 Fab H and L variable regions were generated from PDBs 5VAG^51^ and 3H42 (ref. ^52^), respectively. This structure was built manually in Coot 0.9.6 (ref. ^53^) and refined in Namdinator^54^ and PHENIX 1.20.1 (ref. ^55^).

The resulting model, as well as spike protomers from 6XKL^41^ were fitted into 3:1, 3:2, 3:3 S-GSAS/6P:R1-32 Fab and 3:3:3 S-GSAS/6P:R1-32 Fab:ACE2 maps in Chimera. Real-space refinement was carried out iteratively in Coot and PHENIX. Model refinement statistics are summarized in Supplementary Table 2. Interfaces analysis was performed by PISA^56^. Figures were generated in UCSF Chimera^57^.

### Analysis of IGHV1-69-encoded IgH sequences and R1-32-like sequences

To determine the occurrence of R1-32-like antibodies, we analysed nearly 2 billion IgH sequences from 3 previously described datasets^22–24^. These datasets have been deposited in the National Genomics Data Center (https://bigd.big.ac.cn/), China National Center for Bioinformation (CNCB) under accession numbers PRJCA003775 (ref. ^22^), PRJCA003775 (ref. ^23^) and PRJCA003775 (ref. ^24^). Germline gene usage was determined using MIXCR v3.0.3 (ref. ^58^), and IGHV1-69-encoded IgH sequences were extracted for downstream analysis. The germline usage distribution of RBD-targeted mAbs was calculated using data from the COV-AbDab database (http://opig.stats.ox.ac.uk/webapps/covabdab/). R1-32-like IgH sequences were defined as those sequences that utilized the IGHV1-69 and IGHD5-12 gene segments, had identical HCDR3 length to R1-32 (17 residues), and encoded a GYSGYD/G motif or 80% HCDR3 identity to either R1-32 or FC08. We further allowed up to 3 putative somatic hypermutations (SHM) in the GYSGYD/G motif.

To determine the clonal expansion of R1-32-like lineages in COVID-19 patients after SARS-CoV-2 infection, we tracked the dynamic of R1-32-like IgH sequencing in a representative patient. The divergence from IGHV1-69 of all IgH sequences was equal to somatic hypermutations, and sequence identity to queried sequence was calculated using the R package Biostrings v2.60.2 (http://bioconductor.org/packages/release/bioc/html/Biostrings.html). All IGHV1-69 IgH sequences were plotted as a function of sequence somatic hypermutations (*x* axis) and sequence identity (*y* axis) to an R1-32-like clone PtQ12_28. A colour gradient indicated sequence density. All visualizations were performed using R software (https://www.r-project.org/). Figure 6a describing antibody sequencing was generated with BioRender.com.

### Reporting summary

Further information on research design is available in the Nature Research Reporting Summary linked to this article.

## Supplementary information

Supplementary Information Supplementary Figs. 1–3, Tables 1–10 and reference.

Reporting Summary

## Data Availability

Cryo-EM density maps for the SARS-CoV-2 S trimer in complex with 1 R1-32 Fab, SARS-CoV-2 S trimer in complex with 2 R1-32 Fabs, SARS-CoV-2 S trimer in complex with 3 R1-32 Fabs, SARS-CoV-2 S trimer in complex with 3 R1-32 Fabs and 3 ACE2, and S RBD:R1-32 Fab:ACE2 binding interface have been deposited in the Electron Microscopy Data Bank (EMDB) with accession codes EMD-33760, EMD-33764, EMD-33766, EMD-33772 and EMD-33748. Related atomic models have been deposited in the Protein Data Bank (PDB) under accession codes 7YDY, 7YE5, 7YE9, 7YEG and 7YDI, respectively. Source data are provided with this paper.

## Source data

Source Data Fig. 1 Statistical source data.
Source Data Fig. 4 Unprocessed western blots.
Source Data Extended Data Fig. 1 Statistical source data.
Source Data Extended Data Fig. 2 Statistical source data.
