# Supplementary material for: SARS-CoV-2 Delta and Omicron variants evade population antibody response by mutations in a single spike epitope
Source: Nat Microbiol. 2022 Sep 23;7(10):1635–49. doi: 10.1038/s41564-022-01235-4 (PMC9519457; doi:10.1038/s41564-022-01235-4)
Supplement: Supplementary file 1 — Supplementary Figs. 1–3, Tables 1–10 and reference. [file 41564_2022_1235_MOESM1_ESM.pdf]

nature microbiology

Article https://doi.org/10.1038/s41564-022-01235-4

SARS-CoV-2 Delta and Omicron variants evade population antibody response by mutations in a single spike epitope

In the format provided by the authors and unedited

Supplementary information for

SARS-CoV-2 Delta and Omicron variants evade population antibody response by mutations in a single spike epitope

This file includes the following items:

Supplementary Figures 1-3

Supplementary Tables 1-10

Supplementary Reference

Supplementary Figures

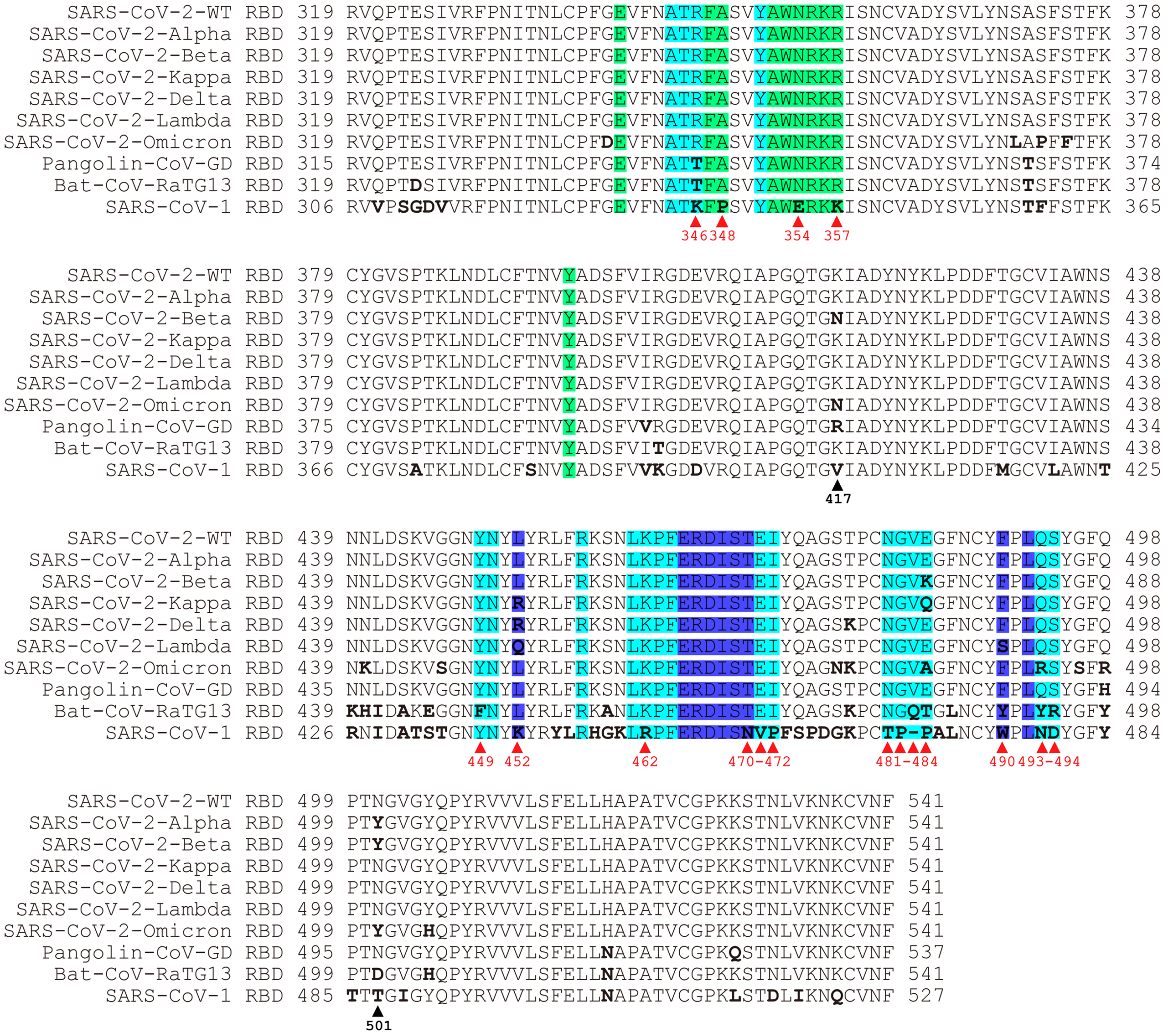

Supplementary Fig. 1 | RBD sequence alignment. Amino acid sequences of RBDs used in this study were aligned. Residues interacting with R1-32-H and R1-32-L are colored in cyan and green respectively, highly buried residues (buried surface area > 70% accessible surface area) are colored blue, exactly the same as in **Fig. 2**. Residues that differ from wild-type SARS-CoV-2 RBD are in bold. Substitutions observed within the R1-32 epitope are indicated by red triangles. Substitutions at 417 and 501 are indicated by black triangles.

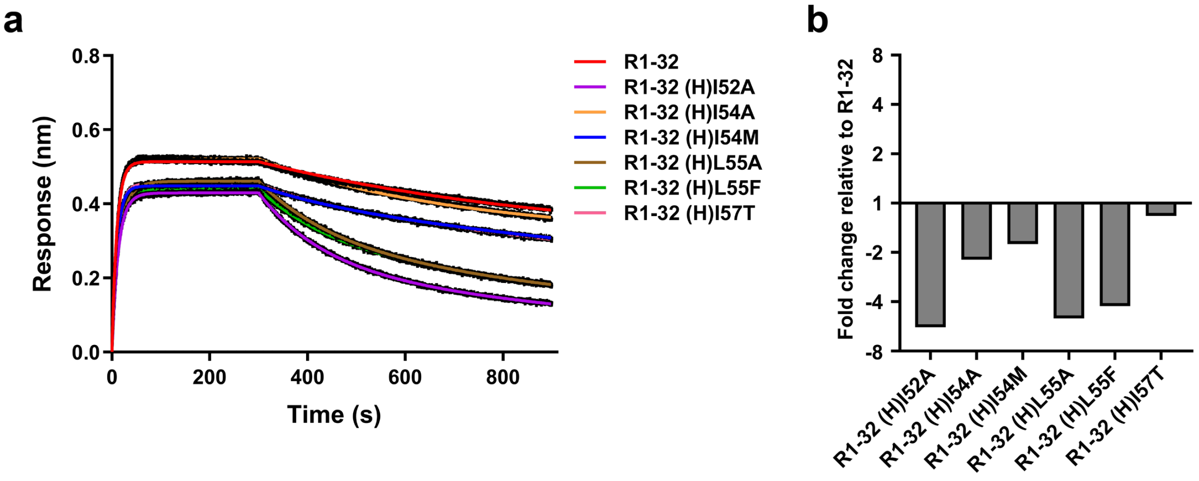

Supplementary Fig. 2 | Polymorphism of VH1-69 HCDR2 loop and its effect on R1-32 antigen binding. a, Sensorgram traces of SARS-CoV-2 wild-type RBD binding (at 200 nM) to immobilized R1-32 and R1-32 variants with substitutions implicated in polymorphism of VH1-69 HCDR2. Kinetic parameters are summarized in **Supplementary Table 10**. **b**, Fold changes of K_D values were normalised to that of R1-32 on a 2^n scale (right panel).

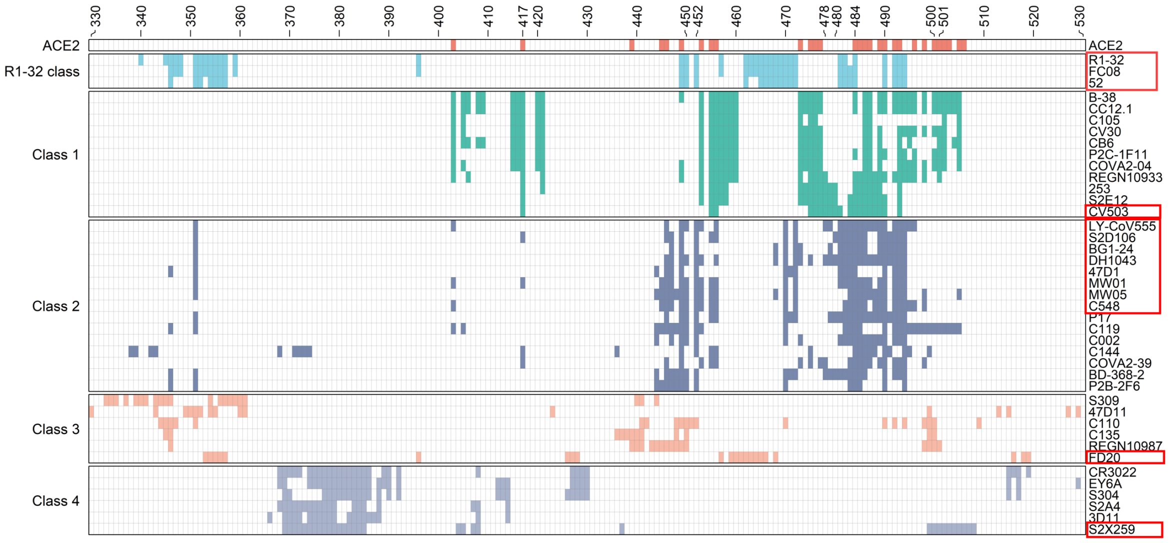

Supplementary Fig. 3 | Classification of RBD targeting antibodies and their epitopes on linear RBD sequence. Epitopes of R1-32, and a list of representative RBD targeting antibodies are compared to the interacting residues of ACE2 on the RBD linear sequence. Epitope residues were identified by PISA with a distance cutoff of 4 Å. The epitope classification followed previous defined antibodies classes[1]. All structurally characterized IGHV1-69 gene encoded antibodies are included and highlighted with red rectangles.

Supplementary Tables

Supplementary Table 1 | Summary of rate constants (k_{on}, k_{off}) and dissociation constants (K_D) for the R1-32 interactions in Fig. 1.

Ligand	Analyte	k_{on} (M^{-1} s^{-1})	k_{off} (s^{-1})	K_D (nM)
R1-32	RBD (WT)	4.72×10^5	3.76×10^{-4}	0.8
	RBD (Alpha)	4.99×10^5	3.54×10^{-4}	0.71
	RBD (Beta)	4.44×10^5	4.56×10^{-3}	10.26
	RBD (Kappa)	1.15×10^5	1.19×10^{-2}	103
	RBD (Delta)	1.78×10^5	1.13×10^{-2}	63.19
	RBD (Lambda)	1.08×10^5	5.04×10^{-2}	467
	RBD (Omicron BA.1)	3.84×10^5	4.36×10^{-4}	1.13
	RBD (Pangolin-CoV-GD)	5.37×10^5	4.20×10^{-4}	0.78
	RBD (Bat-CoV-RaTG13)	2.22×10^5	1.32×10^{-2}	59.13
	RBD (SARS-CoV-1)	-	-	no binding
	spike (WT)	2.86×10^4 (k_{on1})	1.40×10^{-4} (k_{off1})	4.88 (k_{off1}/k_{on1})
		2.11×10^5 (k_{on2})	1.05×10^{-7} (k_{off2})	0.66 (k_{off1}/k_{on2})
				$\leqslant$0.0037 (k_{off2}/k_{on1})
	spike (Alpha)	3.53×10^4 (k_{on1})	7.68×10^{-5} (k_{off1})	2.17 (k_{off1}/k_{on1})
		4.57×10^5 (k_{on2})	3.38×10^{-5} (k_{off2})	0.17 (k_{off1}/k_{on2})
				0.96 (k_{off2}/k_{on1})
				0.074 (k_{off2}/k_{on2})
	spike (Beta)	1.75×10^4 (k_{on1})	3.07×10^{-5} (k_{off1})	1.75 (k_{off1}/k_{on1})
		1.45×10^5 (k_{on2})	1.18×10^{-7} (k_{off2})	0.21 (k_{off1}/k_{on2})
				$\leqslant$0.0067 (k_{off2}/k_{on1})
	spike (Delta)	8.81×10^3 (k_{on1})	2.09×10^{-5} (k_{off1})	2.38 (k_{off1}/k_{on1})
		1.86×10^4 (k_{on2})	$< 1.00\times10^{-7}$ (k_{off2})	1.12 (k_{off1}/k_{on2})
				$<$ 0.011 (k_{off2}/k_{on1})
	spike (Omicron BA.1)	2.43×10^4 (k_{on1})	1.61×10^{-5} (k_{off1})	0.66 (k_{off1}/k_{on1})
		1.80×10^5 (k_{on2})	3.21×10^{-7} (k_{off2})	$\leqslant$0.089 (k_{off1}/k_{on2})

Supplementary Table 2 | Cryo-EM data collection, refinement and validation statistics.

	S-GSAS/6P : Fab 3 : 1 (EMD-33760, PDB 7YDY)	S-GSAS/6P : Fab 3 : 2 (EMD-33764, PDB 7YE5)	S-GSAS/6P : Fab 3 : 3 (EMD-33766, PDB 7YE9)	S-GSAS/6P : Fab : ACE2 3 : 3 : 3 (EMD-33772, PDB 7YEG)	RBD-Fab-ACE2 (EMD-33748, PDB 7YDI)
**Data collection and processing**					
Magnification	45000	45000	45000	45000	45000
Voltage (kV)	200	200	200	200	200
Electron exposure (e^{-}/Å^2)	63	63	63	63	63
Defocus range (μm)	0.8-2.5	0.8-2.5	0.8-2.5	0.8-2.5	0.8-2.5
Pixel size (Å)	0.88	0.88	0.88	0.88	0.88
Movies (no.)	6989	10335	3346	13009	13009
Initial particle images (no.)	619803	1020000	400197	1200393	1200393
Symmetry imposed	*C1*	*C1*	*C3*	*C3*	*C1*
Final particle images (no.)	91441	51351	56902	203698	150762
Map resolution (Å)	4.75	6.75	4.17	3.73	3.98
FSC threshold	0.143	0.143	0.143	0.143	0.143
Map resolution range (Å)	4.11-15.65	5.39-15.93	3.72-14.21	3.33-11.78	3.79-7.15
**Refinement**					
Initial model used	PDB 6XKL	PDB 6XKL	PDB 6XKL	PDB 6XKL	PDB 6M0J
Model resolution (Å)	8.35	9.01	8.17	8.94	4.40
FSC threshold	0.5	0.5	0.5	0.5	0.5
Map sharpening *B* factor (Å^2)	-163	-287.5	-106.5	-97.5	-83.3
Model composition					
Non-hydrogen atoms	28778	32002	35226	49992	8166
Protein residues	3638	4078	4518	6306	1023
Ligands	36	36	36	48	5
B factors (Å^2)					
Protein	354.36	520.90	253.46	416.52	215.3
Ligand	305.08	555.22	175.66	284.90	167.15
R.m.s. deviations					
Bond lengths (Å)	0.001	0.002	0.002	0.002	0.002
Bond angles (°)	0.379	0.397	0.443	0.430	0.449
**Validation**					
MolProbity score	1.40	1.41	1.44	1.41	1.31
Clashscore	4.55	4.74	5.02	4.59	5.70
Poor rotamers (%)	0.03	0.09	0.15	0.04	0.80
Ramachandran plot					
Favored (%)	97.05	97.07	96.98	96.97	98.23
Allowed (%)	2.95	2.93	3.02	3.03	1.77
Disallowed (%)	0.00	0.00	0.00	0.00	0.00

Supplementary Table 3 | Summary of rate constants (k_{on}, k_{off}) and dissociation constants (K_D) for the antibody interactions in Fig. 3.

	RBD			S-R/PP			S-R			S-R/x2			S-R/x3		
	k_{on} (M^{-1} s^{-1})	k_{off} (s^{-1})	K_D (nM)	k_{on} (M^{-1} s^{-1})	k_{off} (s^{-1})	K_D (nM)	k_{on} (M^{-1} s^{-1})	k_{off} (s^{-1})	K_D (nM)	k_{on} (M^{-1} s^{-1})	k_{off} (s^{-1})	K_D (nM)	k_{on} (M^{-1} s^{-1})	k_{off} (s^{-1})	K_D (nM)
**R1-32**	4.72×10^{5}	3.76×10^{-4}	0.8	2.86×10^{4} (k_{on1})	1.40×10^{-4} (k_{off1})	4.88 (k_{off1}/k_{on1})	1.52×10^{4} (k_{on1})	3.03×10^{-5} (k_{off1})	1.99 (k_{off1}/k_{on1})	9.56×10^{3} (k_{on1})	1.92×10^{-5} (k_{off1})	2 (k_{off1}/k_{on1})	1.72×10^{4} (k_{on1})	8.59×10^{-6} (k_{off1})	0.5 (k_{off1}/k_{on1})
				2.11×10^{5} (k_{on2})	1.05×10^{-7} (k_{off2})	0.66 (k_{off1}/k_{on2})	9.58×10^{4} (k_{on2})	$< 1.00\times10^{-7}$ (k_{off2})	0.32 (k_{off1}/k_{on2})	1.02×10^{4} (k_{on2})	$< 1.00\times10^{-7}$ (k_{off2})	1.88 (k_{off1}/k_{on2})	2.49×10^{4} (k_{on2})	$< 1.00\times10^{-7}$ (k_{off2})	0.34 (k_{off1}/k_{on2})
						$\leqslant 0.0037$ (k_{off2}/k_{on1})			< 0.0066 (k_{off2}/k_{on1})			< 0.01 (k_{off2}/k_{on1})			< 0.0058 (k_{off2}/k_{on1})
**B38 (Class 1)**	3.12×10^{5}	1.62×10^{-2}	51.94	-	-	weak binding	-	-	no binding	-	-	no binding	-	-	no binding
**rmAb23 (Class 1)**	1.21×10^{5}	1.45×10^{-2}	119.11	1.85×10^{4} (k_{on1})	2.83×10^{-4} (k_{off1})	15.33 (k_{off1}/k_{on1})	-	-	weak binding	-	-	no binding	-	-	no binding
				2.22×10^{4} (k_{on2})	$< 1.00\times10^{-7}$ (k_{off2})	12.73 (k_{off1}/k_{on2})									
						< 0.0054 (k_{off2}/k_{on1})									
**C144 (Class 2)**	5.91×10^{4}	3.08×10^{-3}	52.18	2.09×10^{4} (k_{on1})	3.78×10^{-4} (k_{off1})	18.11 (k_{off1}/k_{on1})	2.29×10^{4} (k_{on1})	4.11×10^{-4} (k_{off1})	17.98 (k_{off1}/k_{on1})	-	-	weak binding	-	-	weak binding
				2.27×10^{4} (k_{on2})	$< 1.00\times10^{-7}$ (k_{off2})	16.66 (k_{off1}/k_{on2})	2.57×10^{4} (k_{on2})	$< 1.00\times10^{-7}$ (k_{off2})	15.98 (k_{off1}/k_{on2})						
						< 0.0048 (k_{off2}/k_{on1})			< 0.0044 (k_{off2}/k_{on1})						
**S309 (Class 3)**	4.29×10^{4}	2.78×10^{-4}	6.47	1.67×10^{4} (k_{on1})	7.70×10^{-5} (k_{off1})	4.61 (k_{off1}/k_{on1})	1.63×10^{4} (k_{on1})	5.53×10^{-5} (k_{off1})	3.39 (k_{off1}/k_{on1})	2.43×10^{3} (k_{on1})	1.22×10^{-4} (k_{off1})	50.16 (k_{off1}/k_{on1})	1.61×10^{4} (k_{on1})	4.66×10^{-5} (k_{off1})	2.89 (k_{off1}/k_{on1})
				7.18×10^{4} (k_{on2})	$< 1.00\times10^{-7}$ (k_{off2})	1.07 (k_{off1}/k_{on2})	3.64×10^{4} (k_{on2})	$< 1.00\times10^{-7}$ (k_{off2})	1.52 (k_{off1}/k_{on2})	2.01×10^{4} (k_{on2})	$< 1.00\times10^{-7}$ (k_{off2})	6.07 (k_{off1}/k_{on2})	2.21×10^{4} (k_{on2})	$< 1.00\times10^{-7}$ (k_{off2})	2.11 (k_{off1}/k_{on2})
						< 0.006 (k_{off2}/k_{on1})			< 0.0061 (k_{off2}/k_{on1})			< 0.041 (k_{off2}/k_{on1})			< 0.0062 (k_{off2}/k_{on1})
**CR3022 (Class 4)**	4.78×10^{5}	1.27×10^{-2}	26.64	1.50×10^{4} (k_{on1})	1.02×10^{-4} (k_{off1})	6.79 (k_{off1}/k_{on1})	1.00×10^{4} (k_{on1})	1.27×10^{-5} (k_{off1})	1.27 (k_{off1}/k_{on1})	-	-	no binding	-	-	no binding
				3.98×10^{4} (k_{on2})	$< 1.00\times10^{-7}$ (k_{off2})	2.56 (k_{off1}/k_{on2})	1.09×10^{4} (k_{on2})	$< 1.00\times10^{-7}$ (k_{off2})	1.17 (k_{off1}/k_{on2})						
						< 0.0067 (k_{off2}/k_{on1})			< 0.01 (k_{off2}/k_{on1})						

Supplementary Table 4 | Patient information.

Donor	Gender	Age	Hospitalization date	Symptom onset day	Sign of recovery date	Discharge date	Disease severity	Symptom	Days of hospital stay	Sample collection time (Days post symptom onset)
PtF	Female	33	Jan 26, 2020	Jan 25, 2020	Feb 6, 2020	Feb 19, 2020	Severe	Fever, cough, shortness of breath, poor appetite	24	7
PtK	Female	51	Jan 21, 2020	Jan 17, 2020	Feb 1, 2020	Feb 13, 2020	Severe	Fever, cough, poor appetite	23	17
PtH	Male	53	Jan 21, 2020	Jan 19, 2020	Jan 27, 2020	Feb 13, 2020	Mild	Fever, cough	23	15
PtS	Female	68	Jan 26, 2020	Jan 25, 2020	Feb 6, 2020	Feb 13, 2020	Mild	Fever	18	7
PtZ	Male	70	Jan 28, 2020	Jan 20, 2020	Feb 7, 2020	Feb 13, 2020	Mild	Fever, cough, chest distress, poor appetite	16	12
PtL	Female	81	Jan 22, 2020	Jan 12, 2020	Jan 30, 2020	Feb 6, 2020	Severe	Fever, poor appetite	15	22

Supplementary Table 5 | Kinetic parameters of the interactions between the six isolated antibodies and SARS-CoV-2 RBD (Extended Data Fig. 1).

Ligand	Analyte	k_{on} (M^{-1} s^{-1})	k_{off} (s^{-1})	K_D (nM)
R1-26	RBD-WT	4.60×10^{5}	1.77×10^{-3}	3.84
R1-30	RBD-WT	1.17×10^{6}	4.13×10^{-2}	35.38
R1-32	RBD-WT	4.72×10^{5}	3.76×10^{-4}	0.8
R2-3	RBD-WT	8.03×10^{5}	1.07×10^{-2}	13.28
R2-6	RBD-WT	4.90×10^{5}	1.12×10^{-2}	22.8
R2-7	RBD-WT	1.50×10^{6}	9.32×10^{-2}	62.2

Supplementary Table 6 | Summary of rate constants (k_{on}, k_{off}) and dissociation constants (K_D) for the interactions between R1-32 and RBD mutants in Extended Data Fig. 5.

Ligand	Analyte	k_{on} (M^{-1} s^{-1})	k_{off} (s^{-1})	K_D (nM)
R1-32	RBD-WT	5.33×10^{5}	1.49×10^{-3}	2.8
	RBD-Y351F	4.55×10^{5}	1.87×10^{-3}	4.12
	RBD-K417N	4.76×10^{5}	5.36×10^{-4}	1.13
	RBD-L452R	1.66×10^{5}	1.07×10^{-2}	64.52
	RBD-L452Q	2.10×10^{5}	7.33×10^{-3}	34.97
	RBD-K462R	5.09×10^{5}	1.14×10^{-3}	2.25
	RBD-I468V	4.47×10^{5}	2.50×10^{-3}	5.6
	RBD-I468T	4.28×10^{5}	1.14×10^{-2}	26.61
	RBD-T470N	2.81×10^{5}	6.61×10^{-3}	23.51
	RBD-E471V	4.97×10^{5}	1.65×10^{-3}	3.32
	RBD-I472P	2.94×10^{5}	5.74×10^{-3}	19.52
	RBD-I472L	5.09×10^{5}	1.51×10^{-3}	2.97
	RBD-I472V	4.34×10^{5}	3.07×10^{-3}	7.07
	RBD-I472T	2.98×10^{5}	4.66×10^{-3}	15.63
	RBD-loop-R	3.83×10^{5}	4.65×10^{-3}	12.14
	RBD-T478K	4.38×10^{5}	9.27×10^{-4}	2.12
	RBD-E484K	3.67×10^{5}	1.30×10^{-3}	3.55
	RBD-E484Q	3.91×10^{5}	1.16×10^{-3}	2.96
	RBD-F490W	2.16×10^{5}	9.49×10^{-3}	43.89
	RBD-F490S	2.42×10^{5}	6.21×10^{-3}	25.71
	RBD-Alpha	4.11×10^{5}	1.95×10^{-3}	4.74
	RBD-Beta	3.09×10^{5}	3.65×10^{-3}	11.84
	RBD-Kappa	1.21×10^{5}	1.07×10^{-2}	88.37
	RBD-Delta	2.11×10^{5}	1.02×10^{-2}	48.5
	RBD-Lambda	5.07×10^{4}	6.01×10^{-2}	1186

Supplementary Table 7 | Summary of rate constants (k_{on}, k_{off}) and dissociation constants (K_D) for the VH1-69 antibody interactions in Extended Data Fig. 6.

	S-R			S-R/Delta			S-R/x3			S-R/x3/Delta		
	k_{on} (M^{-1} s^{-1})	k_{off} (s^{-1})	K_D (nM)	k_{on} (M^{-1} s^{-1})	k_{off} (s^{-1})	K_D (nM)	k_{on} (M^{-1} s^{-1})	k_{off} (s^{-1})	K_D (nM)	k_{on} (M^{-1} s^{-1})	k_{off} (s^{-1})	K_D (nM)
**R1-32**	1.52×10^{4} (k_{on1})	3.03×10^{-5} (k_{off1})	1.99 (k_{off1}/k_{on1})	8.81×10^{3} (k_{on1})	2.09×10^{-5} (k_{off1})	2.38 (k_{off1}/k_{on1})	1.72×10^{4} (k_{on1})	8.59×10^{-6} (k_{off1})	0.5 (k_{off1}/k_{on1})	1.01×10^{4} (k_{on1})	7.65×10^{-6} (k_{off1})	0.76 (k_{off1}/k_{on1})
	9.58×10^{4} (k_{on2})	$< 1.00\times10^{-7}$ (k_{off2})	0.32 (k_{off1}/k_{on2})	1.86×10^{4} (k_{on2})	$< 1.00\times10^{-7}$ (k_{off2})	1.12 (k_{off1}/k_{on2})	2.49×10^{4} (k_{on2})	$< 1.00\times10^{-7}$ (k_{off2})	0.34 (k_{off1}/k_{on2})	1.11×10^{4} (k_{on2})	$< 1.00\times10^{-7}$ (k_{off2})	0.69 (k_{off1}/k_{on2})
			< 0.0066 (k_{off2}/k_{on1})			< 0.011 (k_{off2}/k_{on1})			< 0.0058 (k_{off2}/k_{on1})			< 0.0099 (k_{off2}/k_{on1})
**FC08 (R1-32 related Ab)**	1.76×10^{4} (k_{on1})	2.01×10^{-7} (k_{off1})	≤ 0.011 (k_{off1}/k_{on1})	1.92×10^{4} (k_{on1})	2.43×10^{-7} (k_{off1})	≤ 0.013 (k_{off1}/k_{on1})	1.30×10^{4} (k_{on1})	7.81×10^{-5} (k_{off1})	6.03 (k_{off1}/k_{on1})	2.47×10^{4} (k_{on1})	1.28×10^{-4} (k_{off1})	5.16 (k_{off1}/k_{on1})
	1.54×10^{5} (k_{on2})	$< 1.00\times10^{-7}$ (k_{off2})		1.26×10^{5} (k_{on2})	$< 1.00\times10^{-7}$ (k_{off2})		2.59×10^{4} (k_{on2})	$< 1.00\times10^{-7}$ (k_{off2})	3.02 (k_{off1}/k_{on2})	2.61×10^{4} (k_{on2})	$< 1.00\times10^{-7}$ (k_{off2})	4.89 (k_{off1}/k_{on2})
									< 0.0077 (k_{off2}/k_{on1})			< 0.004 (k_{off2}/k_{on1})
**52 (R1-32 related Ab)**	2.09×10^{4} (k_{on1})	1.36×10^{-7} (k_{off1})	≤ 0.0065 (k_{off1}/k_{on1})	-	-	no binding	6.35×10^{3} (k_{on1})	3.07×10^{-4} (k_{off1})	48.31 (k_{off1}/k_{on1})	-	-	no binding
	1.59×10^{5} (k_{on2})	$< 1.00\times10^{-7}$ (k_{off2})					1.28×10^{4} (k_{on2})	3.89×10^{-5} (k_{off2})	24.05 (k_{off1}/k_{on2})			
									6.13 (k_{off2}/k_{on1})			
									3.05 (k_{off2}/k_{on2})			
**LY-CoV555 (VH1-69 class 2)**	4.26×10^{4} (k_{on1})	1.57×10^{-5} (k_{off1})	0.37 (k_{off1}/k_{on1})	3.69×10^{4} (k_{on1})	3.98×10^{-4} (k_{off1})	10.79 (k_{off1}/k_{on1})	2.48×10^{4} (k_{on1})	1.40×10^{-4} (k_{off1})	5.64 (k_{off1}/k_{on1})	4.13×10^{4} (k_{on1})	3.28×10^{-3} (k_{off1})	79.41 (k_{off1}/k_{on1})
	3.58×10^{5} (k_{on2})	$< 1.00\times10^{-7}$ (k_{off2})	≤ 0.044 (k_{off1}/k_{on2})	1.96×10^{5} (k_{on2})	4.67×10^{-5} (k_{off2})	2.03 (k_{off1}/k_{on2})	1.46×10^{5} (k_{on2})	2.30×10^{-7} (k_{off2})	0.96 (k_{off1}/k_{on2})	1.67×10^{5} (k_{on2})	2.47×10^{-4} (k_{off2})	19.67 (k_{off1}/k_{on2})
						1.27 (k_{off2}/k_{on1})			≤ 0.0093 (k_{off2}/k_{on1})			5.98 (k_{off2}/k_{on1})
						0.24 (k_{off2}/k_{on2})						1.48 (k_{off2}/k_{on2})
**BG1-24 (VH1-69 class 2)**	3.28×10^{4} (k_{on1})	1.82×10^{-7} (k_{off1})	≤ 0.0056 (k_{off1}/k_{on1})	1.84×10^{4} (k_{on1})	3.58×10^{-6} (k_{off1})	0.19 (k_{off1}/k_{on1})	3.32×10^{4} (k_{on1})	3.46×10^{-5} (k_{off1})	1.04 (k_{off1}/k_{on1})	1.61×10^{4} (k_{on1})	8.90×10^{-6} (k_{off1})	0.55 (k_{off1}/k_{on1})
	3.55×10^{5} (k_{on2})	$< 1.00\times10^{-7}$ (k_{off2})		1.82×10^{5} (k_{on2})	$< 1.00\times10^{-7}$ (k_{off2})	≤ 0.02 (k_{off1}/k_{on2})	2.01×10^{5} (k_{on2})	1.04×10^{-5} (k_{off2})	0.17 (k_{off1}/k_{on2})	1.20×10^{5} (k_{on2})	$< 1.00\times10^{-7}$ (k_{off2})	≤ 0.074 (k_{off1}/k_{on2})
									0.31 (k_{off2}/k_{on1})			
									0.052 (k_{off2}/k_{on2})			
**DH1043 (VH1-69 class 2)**	2.28×10^{4} (k_{on1})	1.11×10^{-7} (k_{off1})	≤ 0.0049 (k_{off1}/k_{on1})	7.92×10^{3} (k_{on1})	3.58×10^{-6} (k_{off1})	0.45 (k_{off1}/k_{on1})	1.79×10^{4} (k_{on1})	1.09×10^{-4} (k_{off1})	6.07 (k_{off1}/k_{on1})	1.98×10^{4} (k_{on1})	6.91×10^{-5} (k_{off1})	3.48 (k_{off1}/k_{on1})
	1.98×10^{5} (k_{on2})	$< 1.00\times10^{-7}$ (k_{off2})		5.38×10^{4} (k_{on2})	$< 1.00\times10^{-7}$ (k_{off2})	≤ 0.067 (k_{off1}/k_{on2})	1.07×10^{5} (k_{on2})	1.67×10^{-7} (k_{off2})	1.01 (k_{off1}/k_{on2})	2.20×10^{4} (k_{on2})	$< 1.00\times10^{-7}$ (k_{off2})	3.14 (k_{off1}/k_{on2})
									≤ 0.0091 (k_{off2}/k_{on1})			< 0.005 (k_{off2}/k_{on1})
**MW01 (VH1-69 class 2)**	2.95×10^{4} (k_{on1})	2.67×10^{-7} (k_{off1})	≤ 0.0091 (k_{off1}/k_{on1})	7.29×10^{3} (k_{on1})	4.19×10^{-5} (k_{off1})	5.75 (k_{off1}/k_{on1})	1.59×10^{4} (k_{on1})	4.89×10^{-5} (k_{off1})	3.09 (k_{off1}/k_{on1})	-	-	no binding
	2.19×10^{5} (k_{on2})	$< 1.00\times10^{-7}$ (k_{off2})		2.05×10^{5} (k_{on2})	$< 1.00\times10^{-7}$ (k_{off2})	0.2 (k_{off1}/k_{on2})	4.68×10^{4} (k_{on2})	$< 1.00\times10^{-7}$ (k_{off2})	1.05 (k_{off1}/k_{on2})			
						< 0.014 (k_{off2}/k_{on1})			< 0.0063 (k_{off2}/k_{on1})			
**MW05 (VH1-69 class 2)**	3.90×10^{4} (k_{on1})	1.00×10^{-7} (k_{off1})	≤ 0.0026 (k_{off1}/k_{on1})	7.34×10^{3} (k_{on1})	2.01×10^{-5} (k_{off1})	2.73 (k_{off1}/k_{on1})	1.99×10^{4} (k_{on1})	2.12×10^{-4} (k_{off1})	10.63 (k_{off1}/k_{on1})	4.30×10^{4} (k_{on1})	3.35×10^{-3} (k_{off1})	77.93 (k_{off1}/k_{on1})
	3.77×10^{5} (k_{on2})	$< 1.00\times10^{-7}$ (k_{off2})		2.46×10^{5} (k_{on2})	$< 1.00\times10^{-7}$ (k_{off2})	≤ 0.081 (k_{off1}/k_{on2})	1.71×10^{5} (k_{on2})	$< 1.00\times10^{-7}$ (k_{off2})	1.24 (k_{off1}/k_{on2})	1.97×10^{5} (k_{on2})	$< 1.00\times10^{-7}$ (k_{off2})	17.01 (k_{off1}/k_{on2})
									< 0.005 (k_{off2}/k_{on1})			< 0.0023 (k_{off2}/k_{on1})

Supplementary Table 8 | Summary of rate constants (k_{on}, k_{off}) and dissociation constants (K_D) for the antibody interactions in Extended Data Fig. 9.

Ligand	Analyte	k_{on} (M^{-1} s^{-1})	k_{off} (s^{-1})	K_D (nM)	Ligand	Analyte	k_{on} (M^{-1} s^{-1})	k_{off} (s^{-1})	K_D (nM)	Ligand	Analyte	k_{on} (M^{-1} s^{-1})	k_{off} (s^{-1})	K_D (nM)
**R1-32**	RBD-WT	5.33×10^{5}	1.49×10^{-3}	2.8	**FC08 (R1-32 related Ab)**	RBD-WT	7.97×10^{5}	2.80×10^{-4}	0.35	**52 (R1-32 related Ab)**	RBD-WT	8.57×10^{5}	1.02×10^{-1}	119
	RBD-K417N	4.76×10^{5}	5.36×10^{-4}	1.13		RBD-K417N	6.78×10^{5}	1.02×10^{-4}	0.15		RBD-K417N	1.77×10^{6}	1.25×10^{-3}	0.71
	RBD-N501Y	4.11×10^{5}	1.95×10^{-3}	4.74		RBD-N501Y	1.69×10^{6}	2.50×10^{-4}	0.15		RBD-N501Y	1.72×10^{6}	2.61×10^{-1}	152
	RBD-E484K	3.67×10^{5}	1.30×10^{-3}	3.55		RBD-E484K	6.63×10^{5}	6.91×10^{-4}	1.04		RBD-E484K	2.45×10^{6}	1.36×10^{-2}	5.54
	RBD-E484Q	3.91×10^{5}	1.16×10^{-3}	2.96		RBD-E484Q	7.15×10^{5}	5.20×10^{-4}	0.73		RBD-E484Q	1.24×10^{6}	5.66×10^{-2}	45.54
	RBD-L452R	1.66×10^{5}	1.07×10^{-2}	64.52		RBD-L452R	3.05×10^{5}	7.73×10^{-3}	25.29		RBD-L452R	-	-	no binding
	RBD-T478K	4.38×10^{5}	9.27×10^{-4}	2.12		RBD-T478K	7.63×10^{5}	2.81×10^{-4}	0.37		RBD-T478K	1.07×10^{6}	4.30×10^{-4}	0.4
	RBD-F490S	2.42×10^{5}	6.21×10^{-3}	25.71		RBD-F490S	6.02×10^{5}	4.49×10^{-3}	7.46		RBD-F490S	-	-	no binding
	RBD-Beta	3.09×10^{5}	3.65×10^{-3}	11.84		RBD-Beta	1.84×10^{6}	1.34×10^{-3}	0.73		RBD-Beta	1.30×10^{6}	1.34×10^{-1}	103
	RBD-Kappa	1.21×10^{5}	1.07×10^{-2}	88.37		RBD-Kappa	3.80×10^{5}	9.64×10^{-3}	25.4		RBD-Kappa	-	-	no binding
	RBD-Delta	2.11×10^{5}	1.02×10^{-2}	48.5		RBD-Delta	3.39×10^{5}	9.44×10^{-3}	27.82		RBD-Delta	-	-	no binding
**LY-CoV555 (VH1-69 class 2)**	RBD-WT	5.89×10^{5}	3.39×10^{-3}	5.76	**BG1-24 (VH1-69 class 2)**	RBD-WT	5.77×10^{5}	3.12×10^{-4}	0.54	**DH1043 (VH1-69 class 2)**	RBD-WT	3.43×10^{5}	1.86×10^{-3}	5.42
	RBD-K417N	5.49×10^{5}	1.92×10^{-3}	3.51		RBD-K417N	4.81×10^{5}	2.20×10^{-4}	0.46		RBD-K417N	3.19×10^{5}	1.97×10^{-3}	6.18
	RBD-N501Y	7.93×10^{5}	3.52×10^{-3}	4.44		RBD-N501Y	1.36×10^{6}	4.88×10^{-4}	0.36		RBD-N501Y	4.41×10^{5}	9.00×10^{-4}	2.04
	RBD-E484K	-	-	no binding		RBD-E484K	-	-	no binding		RBD-E484K	-	-	no binding
	RBD-E484Q	-	-	no binding		RBD-E484Q	-	-	no binding		RBD-E484Q	-	-	no binding
	RBD-L452R	6.35×10^{4}	1.56×10^{-2}	246		RBD-L452R	2.22×10^{5}	5.31×10^{-3}	23.49		RBD-L452R	1.12×10^{5}	6.02×10^{-3}	53.92
	RBD-T478K	3.90×10^{5}	1.86×10^{-3}	4.76		RBD-T478K	5.37×10^{5}	3.13×10^{-4}	0.58		RBD-T478K	3.46×10^{5}	3.36×10^{-3}	9.71
	RBD-F490S	2.34×10^{5}	1.21×10^{-1}	516		RBD-F490S	-	-	no binding		RBD-F490S	-	-	no binding
	RBD-Beta	-	-	no binding		RBD-Beta	-	-	no binding		RBD-Beta	-	-	no binding
	RBD-Kappa	-	-	no binding		RBD-Kappa	-	-	no binding		RBD-Kappa	-	-	no binding
	RBD-Delta	6.20×10^{4}	2.01×10^{-2}	324		RBD-Delta	1.55×10^{5}	5.48×10^{-3}	35.39		RBD-Delta	5.43×10^{4}	6.71×10^{-3}	123
**MW01 (VH1-69 class 2)**	RBD-WT	2.60×10^{5}	2.74×10^{-2}	106	**MW05 (VH1-69 class 2)**	RBD-WT	2.57×10^{5}	1.64×10^{-4}	0.64	**C144 (Non-VH1-69 class 2)**	RBD-WT	6.50×10^{4}	1.49×10^{-2}	229
	RBD-K417N	5.59×10^{5}	4.64×10^{-4}	0.83		RBD-K417N	1.55×10^{5}	5.27×10^{-4}	3.41		RBD-K417N	8.24×10^{4}	1.18×10^{-2}	143
	RBD-N501Y	3.17×10^{5}	1.60×10^{-2}	50.68		RBD-N501Y	6.89×10^{5}	1.15×10^{-3}	1.66		RBD-N501Y	8.21×10^{4}	4.44×10^{-2}	541
	RBD-E484K	-	-	no binding		RBD-E484K	-	-	no binding		RBD-E484K	-	-	no binding
	RBD-E484Q	-	-	no binding		RBD-E484Q	-	-	no binding		RBD-E484Q	-	-	no binding
	RBD-L452R	-	-	no binding		RBD-L452R	1.80×10^{5}	1.05×10^{-1}	582		RBD-L452R	7.99×10^{4}	1.45×10^{-2}	182
	RBD-T478K	4.45×10^{5}	1.88×10^{-2}	42.3		RBD-T478K	2.51×10^{5}	5.17×10^{-4}	2.06		RBD-T478K	8.17×10^{4}	1.22×10^{-2}	150
	RBD-F490S	-	-	no binding		RBD-F490S	-	-	no binding		RBD-F490S	-	-	no binding
	RBD-Beta	-	-	no binding		RBD-Beta	-	-	no binding		RBD-Beta	-	-	no binding
	RBD-Kappa	-	-	no binding		RBD-Kappa	-	-	no binding		RBD-Kappa	-	-	no binding
	RBD-Delta	-	-	no binding		RBD-Delta	1.61×10^{5}	1.45×10^{-1}	906		RBD-Delta	8.95×10^{4}	1.33×10^{-2}	148

Supplementary Table 9 | Summary of rate constants (k_{on}, k_{off}) and dissociation constants (K_D) for the antibody interactions in Extended Data Fig. 10.

Ligand	Analyte	k_{on} (M^{-1} s^{-1})	k_{off} (s^{-1})	K_D (nM)	Ligand	Analyte	k_{on} (M^{-1} s^{-1})	k_{off} (s^{-1})	K_D (nM)
**R1-32**	RBD-WT	5.33×10^{5}	1.49×10^{-3}	2.8	**C963**	RBD-WT	7.09×10^{5}	5.93×10^{-4}	0.84
	RBD-L452R	1.66×10^{5}	1.07×10^{-2}	64.52		RBD-L452R	1.10×10^{5}	2.13×10^{-3}	19.34
	RBD-L452Q	2.10×10^{5}	7.33×10^{-3}	34.97		RBD-L452Q	2.79×10^{5}	3.18×10^{-4}	1.14
	RBD-E484K	3.67×10^{5}	1.30×10^{-3}	3.55		RBD-E484K	3.05×10^{5}	6.10×10^{-5}	0.2
	RBD-E484Q	3.91×10^{5}	1.16×10^{-3}	2.96		RBD-E484Q	4.96×10^{5}	1.86×10^{-4}	0.37
	RBD-F490W	2.16×10^{5}	9.49×10^{-3}	43.89		RBD-F490W	4.33×10^{5}	4.23×10^{-4}	0.98
	RBD-F490S	2.42×10^{5}	6.21×10^{-3}	25.71		RBD-F490S	4.94×10^{5}	3.34×10^{-4}	0.68
	RBD-Beta	3.09×10^{5}	3.65×10^{-3}	11.84		RBD-Beta	4.78×10^{5}	2.12×10^{-4}	0.44
	RBD-Kappa	1.21×10^{5}	1.07×10^{-2}	88.37		RBD-Kappa	9.42×10^{4}	3.90×10^{-3}	41.39
	RBD-Delta	2.11×10^{5}	1.02×10^{-2}	48.5		RBD-Delta	1.09×10^{5}	1.82×10^{-3}	16.65
	RBD-Lambda	5.07×10^{4}	6.01×10^{-2}	1186		RBD-Lambda	1.58×10^{5}	4.73×10^{-3}	29.85
**C978**	RBD-WT	5.31×10^{5}	5.24×10^{-4}	0.99	**C941**	RBD-WT	1.53×10^{5}	1.17×10^{-4}	0.77
	RBD-L452R	4.76×10^{4}	5.13×10^{-3}	108		RBD-L452R	2.89×10^{4}	2.56×10^{-4}	8.83
	RBD-L452Q	1.48×10^{5}	1.20×10^{-3}	8.1		RBD-L452Q	4.87×10^{4}	9.30×10^{-5}	1.91
	RBD-E484K	1.95×10^{5}	2.86×10^{-4}	1.46		RBD-E484K	1.09×10^{5}	9.54×10^{-5}	0.88
	RBD-E484Q	3.76×10^{5}	4.96×10^{-4}	1.32		RBD-E484Q	1.37×10^{5}	9.97×10^{-5}	0.73
	RBD-F490W	2.11×10^{5}	3.78×10^{-3}	17.96		RBD-F490W	8.61×10^{4}	1.27×10^{-4}	1.48
	RBD-F490S	2.26×10^{5}	7.67×10^{-4}	3.39		RBD-F490S	6.76×10^{4}	1.08×10^{-4}	1.6
	RBD-Beta	3.10×10^{5}	5.86×10^{-4}	1.89		RBD-Beta	1.80×10^{5}	1.21×10^{-4}	0.67
	RBD-Kappa	3.18×10^{4}	4.90×10^{-3}	154		RBD-Kappa	3.22×10^{4}	2.24×10^{-4}	6.96
	RBD-Delta	4.20×10^{4}	7.35×10^{-3}	175		RBD-Delta	3.17×10^{4}	2.36×10^{-4}	7.46
	RBD-Lambda	4.28×10^{4}	2.07×10^{-3}	48.4		RBD-Lambda	2.83×10^{4}	4.29×10^{-4}	15.17
**C091**	RBD-WT	3.46×10^{5}	3.37×10^{-3}	9.75	**C092**	RBD-WT	6.42×10^{5}	1.03×10^{-4}	0.16
	RBD-L452R	6.53×10^{3}	2.07×10^{-1}	31675		RBD-L452R	1.99×10^{5}	2.90×10^{-5}	0.15
	RBD-L452Q	5.49×10^{4}	1.84×10^{-2}	334		RBD-L452Q	3.02×10^{5}	3.64×10^{-5}	0.12
	RBD-E484K	2.13×10^{5}	2.11×10^{-3}	9.91		RBD-E484K	3.07×10^{5}	3.00×10^{-5}	0.098
	RBD-E484Q	2.62×10^{5}	2.83×10^{-3}	10.81		RBD-E484Q	4.73×10^{5}	7.60×10^{-5}	0.16
	RBD-F490W	1.04×10^{5}	1.77×10^{-2}	170		RBD-F490W	3.83×10^{5}	7.73×10^{-5}	0.2
	RBD-F490S	2.33×10^{5}	6.95×10^{-3}	29.86		RBD-F490S	3.16×10^{5}	7.30×10^{-5}	0.23
	RBD-Beta	2.35×10^{5}	8.84×10^{-3}	37.57		RBD-Beta	4.82×10^{5}	7.14×10^{-5}	0.15
	RBD-Kappa	1.09×10^{4}	2.03×10^{-1}	18554		RBD-Kappa	1.81×10^{5}	5.06×10^{-5}	0.28
	RBD-Delta	1.24×10^{4}	1.49×10^{-1}	11990		RBD-Delta	2.09×10^{5}	2.55×10^{-5}	0.12
	RBD-Lambda	1.71×10^{4}	7.70×10^{-2}	4508		RBD-Lambda	1.43×10^{5}	9.78×10^{-5}	0.68
**C807**	RBD-WT	5.47×10^{5}	4.31×10^{-4}	0.79	**C832**	RBD-WT	3.87×10^{5}	2.46×10^{-4}	0.63
	RBD-L452R	3.16×10^{5}	1.46×10^{-3}	4.64		RBD-L452R	9.30×10^{4}	5.34×10^{-3}	57.39
	RBD-L452Q	3.24×10^{5}	1.96×10^{-3}	6.04		RBD-L452Q	1.71×10^{5}	8.08×10^{-4}	4.73
	RBD-E484K	2.66×10^{5}	6.70×10^{-5}	0.25		RBD-E484K	1.97×10^{5}	6.67×10^{-5}	0.33
	RBD-E484Q	3.82×10^{5}	1.83×10^{-4}	0.48		RBD-E484Q	2.96×10^{5}	1.56×10^{-4}	0.53
	RBD-F490W	4.55×10^{5}	3.19×10^{-4}	0.7		RBD-F490W	2.46×10^{5}	1.73×10^{-3}	7.02
	RBD-F490S	3.10×10^{5}	4.41×10^{-4}	1.42		RBD-F490S	2.07×10^{5}	1.10×10^{-4}	0.53
	RBD-Beta	4.48×10^{5}	2.35×10^{-4}	0.52		RBD-Beta	3.13×10^{5}	2.23×10^{-4}	0.71
	RBD-Kappa	2.65×10^{5}	2.63×10^{-3}	9.92		RBD-Kappa	8.73×10^{4}	6.12×10^{-3}	70.14
	RBD-Delta	3.23×10^{5}	1.87×10^{-3}	5.78		RBD-Delta	9.98×10^{4}	6.38×10^{-3}	63.97
	RBD-Lambda	1.10×10^{5}	2.62×10^{-3}	23.81		RBD-Lambda	5.21×10^{4}	6.05×10^{-3}	116

Supplementary Table 10 | Kinetic parameters of R1-32 and R1-32 variants binding to SARS-CoV-2 RBD (Supplementary Fig. 2).

Ligand	Analyte	k_{on} (M^{-1} s^{-1})	k_{off} (s^{-1})	K_D (nM)
R1-32	RBD-WT	5.33×10^{5}	1.49×10^{-3}	2.8
R1-32 (H)I52A	RBD-WT	4.27×10^{5}	6.87×10^{-3}	16.09
R1-32 (H)I54A	RBD-WT	4.85×10^{5}	3.03×10^{-3}	6.23
R1-32 (H)I54M	RBD-WT	4.90×10^{5}	2.45×10^{-3}	4.99
R1-32 (H)L55A	RBD-WT	4.66×10^{5}	6.59×10^{-3}	14.13
R1-32 (H)L55F	RBD-WT	5.77×10^{5}	6.89×10^{-3}	11.94
R1-32 (H)I57T	RBD-WT	4.56×10^{5}	1.52×10^{-3}	3.35

Supplementary Reference

1 Barnes, C. O. *et al.* SARS-CoV-2 neutralizing antibody structures inform therapeutic strategies. *Nature* **588**, 682-687, doi:10.1038/s41586-020-2852-1 (2020).
