# Supplementary material for: SARS-CoV-2 Delta and Omicron variants evade population antibody response by mutations in a single spike epitope
Source: Nat Microbiol. 2022 Sep 23;7(10):1635–49. doi: 10.1038/s41564-022-01235-4 (PMC9519457; doi:10.1038/s41564-022-01235-4)
Supplement: Supplementary file 2 — Reporting Summary [file 41564_2022_1235_MOESM2_ESM.pdf]

nature portfolio

Corresponding author(s): Xiaoli Xiong

Last updated by author(s): July 23,2022

Reporting Summary

Nature Portfolio wishes to improve the reproducibility of the work that we publish. This form provides structure for consistency and transparency in reporting. For further information on Nature Portfolio policies, see our Editorial Policies and the Editorial Policy Checklist.

Statistics

For all statistical analyses, confirm that the following items are present in the figure legend, table legend, main text, or Methods section.

n/a	Confirmed	
☐	☒	The exact sample size (n) for each experimental group/condition, given as a discrete number and unit of measurement
☐	☒	A statement on whether measurements were taken from distinct samples or whether the same sample was measured repeatedly
☐	☒	The statistical test(s) used AND whether they are one- or two-sided<br>*Only common tests should be described solely by name; describe more complex techniques in the Methods section.*
☒	☐	A description of all covariates tested
☒	☐	A description of any assumptions or corrections, such as tests of normality and adjustment for multiple comparisons
☐	☒	A full description of the statistical parameters including central tendency (e.g. means) or other basic estimates (e.g. regression coefficient) AND variation (e.g. standard deviation) or associated estimates of uncertainty (e.g. confidence intervals)
☐	☒	For null hypothesis testing, the test statistic (e.g. F, t, r) with confidence intervals, effect sizes, degrees of freedom and P value noted<br>*Give P values as exact values whenever suitable.*
☒	☐	For Bayesian analysis, information on the choice of priors and Markov chain Monte Carlo settings
☒	☐	For hierarchical and complex designs, identification of the appropriate level for tests and full reporting of outcomes
☒	☐	Estimates of effect sizes (e.g. Cohen's d, Pearson's r), indicating how they were calculated

Our web collection on statistics for biologists contains articles on many of the points above.

Software and code

Policy information about availability of computer code

Data collection	SerialEM automated image acquisition software version 3.8.7; ForteBio Octet RED96 system; GatorBio system.
Data analysis	CryoEM data processing : RELION 3.1; MotionCor2 algorithm; Warp 1.0.7.<br>Modelling/structure refinement/visualization : Coot 0.9.6 EL; Namdinator (https://namdinator.au.dk/namdinator/); PHENIX 1.20.1; UCSF Chimera 1.14; PDBe PISA v1.52.<br>Antibody repertoire databases analysis: R statistical software (https://www.r-project.org/); MiXCR v3.0.3; Biostrings v2.60.2.<br>Kinetic constants calculation: ForteBio Octet Data Analysis Software; GatorBio Data Analysis Software.<br>Polyreactivity data analysis: Graphpad Prism 8.0.<br>Sequence alignment: IMGT/V-QUEST (http://imgt.org).

For manuscripts utilizing custom algorithms or software that are central to the research but not yet described in published literature, software must be made available to editors and reviewers. We strongly encourage code deposition in a community repository (e.g. GitHub). See the Nature Portfolio guidelines for submitting code & software for further information.

Data

Policy information about availability of data

All manuscripts must include a data availability statement. This statement should provide the following information, where applicable:

- Accession codes, unique identifiers, or web links for publicly available datasets
- A description of any restrictions on data availability
- For clinical datasets or third party data, please ensure that the statement adheres to our policy

Databases used in this study include antibody repertoire databases: (Niu et al., Frontiers in Immunology. 2020) (https://bigd.big.ac.cn/) under the accession

number: PRJCA003775, (Yan et al., Emerging Microbes infections. 2021) (https://bigd.big.ac.cn/) under the accession number: PRJCA003775, (Zhang et al., Journal of Virology. 2022) (http s://bigd.big.ac.cn/) under the accession number: PRJCA007067; The germline usage distribution of RBD-targeted mAbs was calculated using the data from COV-AbDab database: http://opig.stats.ox.ac.uk/webapps/covabdab/.
The structures of antibodies (FC08, 52, LY-CoV555, S2D106, BG1-24, DH1043, 47D1, MW05, MW01, C548, B38, C144, S309, CR3022) used for analysis in this study are available from PDB (http://www.rcsb.org/) under IDs: 7DX4, 7K9Z, 7L3N, 7R7N, 7M6I, 7LJR, 7MF1, 7DK0, 7DJZ, 7R8O, 7BZ5, 7K90, 6WPS, 6YLA . The structures of spike trimers (Xiong et al., Nature Structural Molecular Biology. 2020) obtained from PDB: 6ZOX, 6ZOZ.
Cryo-EM density maps of the SARS-CoV-2 Spiker trimer in complex with one R1-32 Fab, SARS-CoV-2 Spiker trimer in complex with two R1-32 Fabs, SARS-CoV-2 Spiker trimer in complex with three R1-32 Fabs, SARS-CoV-2 Spiker trimer in complex with three R1-32 Fabs and three ACE2, and 3:3:3 spike protomer: Fab: ACE2 complex binding interface have been deposited at the Electron Microscopy Data Bank with accession codes EMD-33760, EMD-33764, EMD-33766, EMD-33772 and EMD-33748. Related atomic models have been deposited in Protein Data Bank under accession codes 7YDY, 7YE5, 7YE9, 7YEG and 7YDI, respectively.

Field-specific reporting

Please select the one below that is the best fit for your research. If you are not sure, read the appropriate sections before making your selection.

☒ Life sciences ☐ Behavioural & social sciences ☐ Ecological, evolutionary & environmental sciences

For a reference copy of the document with all sections, see nature.com/documents/nr-reporting-summary-flat.pdf

Life sciences study design

All studies must disclose on these points even when the disclosure is negative.

Sample size	For the animal study, there are 5 mice in each group ,which meet the requirement for statistical analysis and is sufficient for a good technical reproducibility. Other assays were performed for duplicates or three replicates, which are also sufficient for a good statistical analysis.
Data exclusions	No data were excluded.
Replication	The replicates were used in the experiments as noted in the figure legends and methods.
Randomization	We randomly divided 15 hACE2 transgenic mice into three groups, each group have 5 mice. All mice are females. For lung tissues analyzed with histological staining and virus titer determination assay, images were selected randomly.
Blinding	The investigators were not blinded to allocation during experiments and outcome assessment. Data collection and analysis were performed by different people, the sample classification were replaced by marks during data analysis.

Reporting for specific materials, systems and methods

We require information from authors about some types of materials, experimental systems and methods used in many studies. Here, indicate whether each material, system or method listed is relevant to your study. If you are not sure if a list item applies to your research, read the appropriate section before selecting a response.

Materials & experimental systems

n/a	Involved in the study	
☐	☒	Antibodies
☐	☒	Eukaryotic cell lines
☒	☐	Palaeontology and archaeology
☐	☒	Animals and other organisms
☐	☒	Human research participants
☒	☐	Clinical data
☒	☐	Dual use research of concern

Methods

n/a	Involved in the study	
☒	☐	ChIP-seq
☒	☐	Flow cytometry
☒	☐	MRI-based neuroimaging

Antibodies

Antibodies used	HRP-labeled Goat Anti-Human IgG(H+L) (Beyotime, cat: A0201, dilution 1:5000);<br>HRP-Mouse Anti-M13 antibody (NBbiolab, cat: S004H, dilution 1:5000);<br>SARS-CoV-2 S2 polyclonal antibody (Sino Biological, cat: 40590-T62, dilution 1:2500);<br>HRP-labeled Goat Anti-Rabbit IgG(H+L) (Beyotime, cat: A0208, dilution 1:1000).<br>SARS-CoV-2 specific antibodies: R1-32, R1-26, R1-30, R2-3, R2-6, R2-7 were isolated and produced in our lab; B38, rmAb23, C144, S309, CR3022, FC08, 52, LY-CoV555, BG1-24, DH1043, MW05, MW01, C963, C978, C941, C091, C092, C807, C832 were prepared in our lab.
Validation	HRP-labeled Goat Anti-Human IgG(H+L), HRP-Mouse Anti-M13 antibody, SARS-CoV-2 S2 polyclonal antibody,HRP-labeled Goat Anti-Rabbit IgG(H+L) were established commercial antibody. The validation of commercially available antibodies used in this study was

described in technical data sheets provided by the manufacturers or on their websites.
Antibodies: R1-32, R1-26, R1-30, R2-3, R2-6, R2-7 were validated in this study. Antibodies: B38 (Wu et al., Science. 2020); rmAb23 (Yan et al., Emerging Microbes infections. 2021); C144 (Barnes et al., Nature. 2020); S309 (Pinto et al., Nature. 2020); CR3022 (Yuan et al., Science. 2020); FC08 (Zhang et al., National Science Review. 2021); 52 (Rujas et al., Nature Communications. 2021); LY-CoV555 (Jones et al., Science Translational Medicine. 2021); BG1-24 (Scheid et al., Cell. 2021); DH1043 (Li et al., Cell. 2021); MW05 and MW01 (Wang et al., Communications biology. 2022); C963, C978, C941 (Wang et al., Nature. 2021); C091 and C092 (Gaebler et al., Nature. 2021); C807 and C832 (Wang Z. et al., Nature. 2021) have described previously and are also validated in this study.

Eukaryotic cell lines

Policy information about cell lines

Cell line source(s)	293T cells: ATCC, CRL-3216;<br>Vero E6 cells: ATCC, CRL-1586;<br>Expi293F: ThermoFisher, cat. A14527;<br>Human ACE2 stably expressing HEK293 cell line (HEK293T-hACE2) have described by previously study (Feng et al., Nature Communications. 2020) and was generated in our lab.
Authentication	All cell lines were frequently checked for the cellular morphologies, growth rates and functions in our lab and were not commonly misidentified.
Mycoplasma contamination	The cell lines were not contaminated by mycoplasma as determined by using the Lonza Mycoplasma Detection Kit.
Commonly misidentified lines (See ICLAC register)	No commonly misidentified cell lines were used.

Animals and other organisms

Policy information about studies involving animals; ARRIVE guidelines recommended for reporting animal research

Laboratory animals	For the SARS-CoV-2 animal experiment, the hACE2-transgenic C57BL/6 mice were provided by Cyagen Biosciences Inc. (cat: C001191). All protocols for animal experiments were approved by the Institutional Animal Care and Use Committees of Guangzhou Institutes of Biomedicine and Health, Chinese Academy of Sciences. All work with live SARS-CoV-2 was conducted in the Biosafety Level 3 (BLS3) Laboratories. 20-weeks old female mice were randomly divided into three groups (5 mice per group). All mice were kept in SPF (specific pathogen free) facilities with controlled temperature (20-26°C), humidity (40-70%) and lighting conditions (12h light/ 12h dark cycles).
Wild animals	The study did not involve wild animals.
Field-collected samples	The study did not involve samples collected from the field.
Ethics oversight	The animal study was approved by Ethics Committee of Guangzhou Institutes of Biomedicine and Health, Chinese Academy of Sciences (IACUC: 2020025).

Note that full information on the approval of the study protocol must also be provided in the manuscript.

Human research participants

Policy information about studies involving human research participants

Population characteristics	The COVID-19 convalescent patients (with both females and males) cared by Guangzhou Eighth People's Hospital, China were selected randomly. The COVID-19 convalescent patients selected showed mild or severe symptoms of COVID-9 when they were infected at the initial stage of SARS-CoV-2 pandemic. Donors are age 33-81. We used the peripheral blood mononuclear cells (PBMCs) of the donors to construct scFv phage libraries.
Recruitment	Patients were recruited and clinically diagnosed at Guangzhou Eighth People's Hospital, China. Study participants were recruited on the random basis. There was no potential self-selection bias or other biases during the selection.
Ethics oversight	Ethics Committee of Guangzhou Eighth People's Hospital (REC ref: AF/SC-02/01.6).

Note that full information on the approval of the study protocol must also be provided in the manuscript.
