# Supplementary figures and images for: SARS-CoV-2 Delta and Omicron variants evade population antibody response by mutations in a single spike epitope
Source: Nat Microbiol. 2022 Sep 23;7(10):1635–49. doi: 10.1038/s41564-022-01235-4 (PMC9519457; doi:10.1038/s41564-022-01235-4)

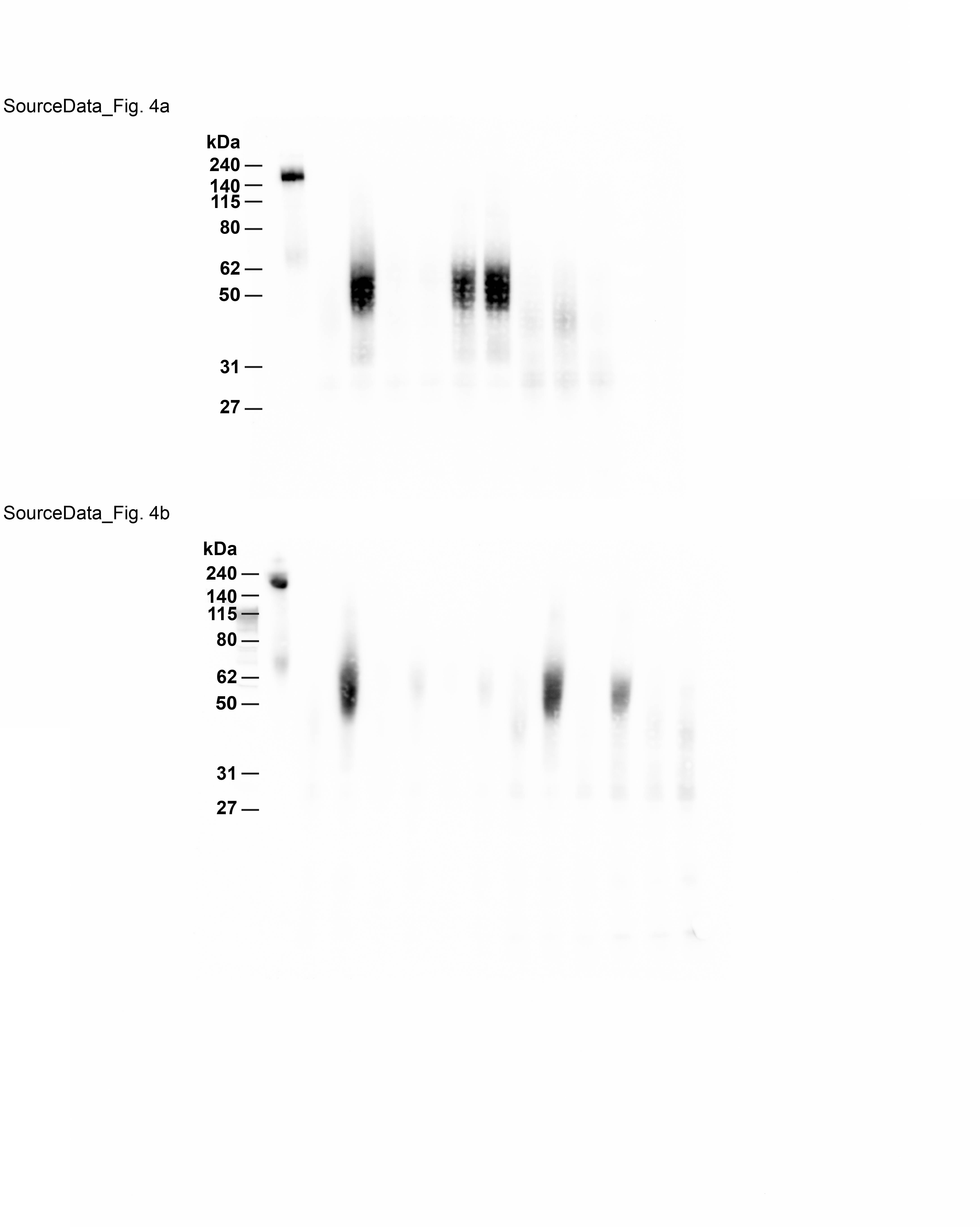

Supplement: Source Data Fig. 4 — Unprocessed western blots. [file 41564_2022_1235_MOESM4_ESM.jpg]
